# The Genomics and Molecular Biology of Natural Killer/T-Cell Lymphoma: Opportunities for Translation

**DOI:** 10.3390/ijms19071931

**Published:** 2018-06-30

**Authors:** Sanjay de Mel, Gwyneth Shook-Ting Soon, Yingting Mok, Tae-Hoon Chung, Anand D. Jeyasekharan, Wee-Joo Chng, Siok-Bian Ng

**Affiliations:** 1 Department of Haematology-Oncology, National University Cancer Institute of Singapore, National University Health System, Singapore 110974, Singapore; sanjay_widanalage@nuhs.edu.sg (S.d.M.); anand_jeyasekharan@nuhs.edu.sg (A.D.J.); 2 Department of Pathology, National University Hospital, National University Health System, Singapore 110974, Singapore; gwyneth.soon@mohh.com.sg (G.S.-T.S.); yingting.mok@mohh.com.sg (Y.M.); 3 Cancer Science Institute of Singapore, National University of Singapore, Singapore 110974, Singapore; hoontaechung@gmail.com; 4 Department of Pathology, Yong Loo Lin School of Medicine, National University of Singapore, Singapore 119074, Singapore

**Keywords:** NK/T-cell lymphoma, genomics, Gene expression profiling (GEP), copy number, epigenetics, immune checkpoint, targeted therapy

## Abstract

Extranodal NK/T-cell lymphoma, nasal type (ENKTL), is an aggressive malignancy with a poor prognosis. While the introduction of L-asparaginase in the treatment of this disease has significantly improved the prognosis, the outcome of patients relapsing after asparaginase-based chemotherapy, which occurs in up to 50% of patients with disseminated disease, remains dismal. There is hence an urgent need for effective targeted therapy especially in the relapsed/refractory setting. Gene expression profiling studies have provided new perspectives on the molecular biology, ontogeny and classification of ENKTL and further identified dysregulated signaling pathways such as Janus associated kinase (/Signal Transducer and activation of transcription (JAK/STAT), Platelet derived growth factor (PDGF), Aurora Kinase and NF-κB, which are under evaluation as therapeutic targets. Copy number analyses have highlighted potential tumor suppressor genes such as PR Domain Zinc Finger Protein 1 (PRDM1) and protein tyrosine phosphatase kappa (PTPRK) while next generation sequencing studies have identified recurrently mutated genes in pro-survival and anti-apoptotic pathways. The discovery of epigenetic dysregulation and aberrant microRNA activity has broadened our understanding of the biology of ENKTL. Importantly, immunotherapy via Programmed Cell Death -1 (PD-1) and Programmed Cell Death Ligand1 (PD-L1) checkpoint signaling inhibition is emerging as an attractive therapeutic strategy in ENKTL. Herein, we present an overview of the molecular biology and genomic landscape of ENKTL with a focus on the most promising translational opportunities.

## 1. Introduction

EBV-associated T-cell or NK-cell lymphoproliferative diseases encompass several disease entities with differing clinical and pathologic findings. The two prototypical entities are extranodal NK/T-cell lymphoma, nasal type (ENKTL) and aggressive NK-cell leukemia (ANKL) [1]. ENKTL is a highly aggressive lymphoma derived from mature NK cells or cytotoxic T cells with a strong association with Epstein Barr Virus (EBV). The disease almost always has an extranodal presentation and classically involves the nasal cavity or upper aerodigestive tract. Other sites of extranasal involvement include skin, soft tissue and gastrointestinal tract. Overall, about 85% of nasal ENKTL are derived from NK cells but only about 50% of those occurring in the extranasal sites are of NK lineage [2]. ENKTL commonly affects adult males and is prevalent in Asia, Mexico, Central or South America but relatively little is known about the precise etiology of this tumor. The strong ethnic predilection seems to suggest a role for the immunogenetic background and environmental factors while the clear association with EBV, irrespective of the ethnic origin of the patients, indicates an important role of the virus in the pathogenesis of the disease [1]. The EBV exists in a clonal episomal form in the tumor cells and shows a type II latency pattern [3]. It is usually of subtype A, with a high frequency of 30–base-pair deletion of the Latent Membrane Protein-1 (*LMP-1)* gene [4,5]. Histologically, the tumor is characterized by diffuse lymphoid infiltration with angiodestructive growth pattern and prominent necrosis. Phenotypically, the neoplastic cells express cCD3; CD2; CD56; cytotoxic markers, such as TIA1, granzyme B and perforin; and EBV. Based on the current WHO classification criteria, the diagnosis of ENKTL requires the expression of cCD3, cytotoxic markers and Epstein-Barr virus encoded RNA (EBER) by in situ hybridization [1].

ENKTL remains a challenging disease to study and the obstacles are mainly a result of the rarity of this disease and limited tissue availability due to prominent necrosis [6]. Furthermore, the distinction of ENKTL from other EBV-associated cytotoxic T- and NK-cell lymphoproliferative diseases, such as ANKL, chronic active EBV infection, systemic EBV-positive T-cell lymphoma, and primary nodal EBV-positive peripheral T-cell lymphoma not otherwise specified (PTCL NOS) can be challenging due to significant overlap in morphology and phenotype [7,8]. The distinction of ENKTL from these closely related entities is heavily dependent on the age at presentation, sites of involvement and T vs. NK-cell lineage. However, as there is no absolutely specific marker for NK-cell origin [2], the diagnosis of an NK-cell malignancy is often dependent on the exclusion of other T-cell derived neoplasm. Moreover, the earlier studies on ENKTL suffered from incomplete workup to distinguish between T vs. NK cell lineage of the cases analyzed, making data interpretation difficult. In addition to the diagnostic challenges, ENKTL is also an aggressive disease in need of more effective treatment modalities. ENKTLs express high levels of the drug exporter p-glycoprotein and, hence, have a poor sensitivity to anthracycline-based chemotherapy [9]. The current standard of care for ENKTL is L-asparaginase based combination chemotherapy with or without radiotherapy. However, outcomes remain poor with a five-year survival of 50% at best for patients with advanced stage disease [10]. Therefore, there is an urgent clinical need to identify effective targeted therapy for this disease.

Molecular profiling is playing an increasingly important role in the diagnosis and classification of lymphomas. The utility of gene expression profiling (GEP) to identify distinct subtypes of diffuse large B-cell lymphoma based on cell of origin is a key example [11]. In terms of guiding management, the presence of the 17p deletion or TP53 mutations are crucial in chronic lymphocytic leukemia [12]. The genomics of T- and NK-cell malignancies have been an active field of research in the recent years. The identification of Ten eleven translocation -2 (TET2), Isocitrate Dehydrogenase (IDH) and DNA methyl transferase 3A (DNMT3A) mutations in peripheral T-cell lymphoma has been a major advance [13,14,15]. In a similar vein, the identification of unique gene expression profiles and dysregulated signaling pathways in ENKTL is providing new insight into the pathogenesis of this disease [6,16]. Despite this progress, current knowledge of the ENKTL genome has yet to produce a meaningful impact on clinical practice. In this review, we aim to highlight the key players in the molecular biology and genetic make-up of ENKTL, with a focus on those with the greatest translational potential.

## 2. Insights from Gene Expression Profiling

### 2.1. Classification

In addition to the delineation of pathways underlying lymphomagenesis, GEP studies have provided new perspectives on the molecular biology, ontogeny and classification of PTCL, including ENKTL. The gene expression profiles of ENKTL cluster together irrespective of NK- or cytotoxic T-cell lineage, supporting the current WHO classification to include tumors of these two lineages in the same lymphoma category [17]. However, the number of cases analyzed is limited [17] and other GEP studies included either mostly ENKTL of NK-cell origin or lacks complete data regarding T vs. NK-cell lineage [18,19,20].

The gene expression profile of ENKTL is distinct from that of PTCL, NOS [17,19] and enriched in genes related to NK-cell activation/survival and NK-cell markers, including Cluster differentiation 244 (*CD244*), *FAS Ligand* (*FASLG*), *Killer Immunoglobulin Receptor 2DL4* (*KIR2DL4*), Killer cell lectin like receptor subfamily D1 (*KLRD1*), SH2 Domain containing protein 1B (*SH2D1B*), Killer cell lectin like receptor subfamily C2 (*KLRC2*) and Neural Cell Adhesion Molecule 1 (*NCAM1)* [21]. Iqbal et al. utilized a molecular classifier based on 84 transcripts, which was able to identify lymphomas originating from NK cells as well as a subset of gamma-delta (γδ) T cells with a very similar molecular signature to NK cells. These γδ PTCLs are non-hepatosplenic in presentation and may be molecularly akin to a subset of ENKTL with γδ T-cell origin, a reflection of the related ontogeny [19]. Using a similar set of transcripts, the authors subsequently demonstrated that the molecular classifier was able to identify 97% (31/32) of ENKTL cases and reclassified 9% (13/150) of PTCL-NOS as ENKTL [18,21]. These cases could then be classified as ENKTL or γδ -PTCL, the latter subgroup showing a high expression of cluster differentiation 3 gamma delta (CD3γδ) and cluster differentiation 3 delta (CD3δ) mRNA as well as transcripts encoding T-cell receptor-gamma (TCRγ) chain. Overall, the classifier for ENKTL is highly specific and the signature for ENKTL is largely shared by tumors of the innate immune system, i.e., NK cells and γδ T cells. The subset of PTCL-NOS belonging to γδ T-cell lymphomas by molecular classification showed clinical outcome similar to ENKTL [19,21].

Using GEP and copy number analysis, it has recently been demonstrated that not all EBV-positive T/NK-cell lymphomas in adults should be classified as ENKTL. Cases presenting with nodal disease are characterized by unique clinicopathologic features, such as old age, lack of nasal involvement, T-cell origin, CD8-positive/CD56-negative phenotype as well as distinctive molecular and copy number signatures, such as the loss of 14q11.2, which correlates with T-cell origin. Since the 14q11.2 locus contains the T-cell receptor alpha constant (TRAC), the loss of 14q11.2 in the nodal cases is likely a reflection of the physiologic rearrangement of the TCR loci as focal loss within the TCR loci can occur during VDJ recombination. Taken together, these features support a distinct molecular biology and suggest that cases presenting with primary nodal disease should be classified separately from ENKTL [22]. This distinction is clinically important as the nodal cases (currently termed “EBV-positive PTCL NOS”) is significantly more aggressive than ENKTL and should be managed differently [22,23]. Other than EBV-positive primary nodal T/NK cell lymphomas, ENKTL also shows significant clinicopathologic and diagnostic overlap with the spectrum of EBV-associated T/NK-cell lymphoproliferative diseases occurring in childhood (TNKLPDC), such as systemic EBV-positive T-cell lymphoma, ANKL and chronic active EBV infection of T/NK cell type [24,25]. Gene set enrichment analysis revealed similarities and subtle differences between TNKLPDC and ENKTL with the former showing a distinctive enrichment of stem cell related genes while ENKTL is enriched for genes over-expressed in advanced solid cancers [26]. This finding of potential cancer stem cell properties in TNKPLDC may have therapeutic implications and may also explain the success of hematopoietic stem cell transplant (HSCT) in the treatment of TNKLPDC and why conventional chemotherapy, without HSCT, is often unsuccessful.

### 2.2. Deregulated Single Genes

#### 2.2.1. Survivin

Survivin belongs to the family of inhibitor of apoptosis proteins, which function to inhibit caspase activation, thereby leading to negative regulation of apoptosis or programmed cell death [27]. Ng and colleagues found overexpression of survivin in 97% of ENKTL cases [20]. The effect of survivin upregulation is that of a pro-survival cellular phenotype, and therefore overexpression of survivin may account for the relative resistance of this tumor to anti-cancer therapy. In vitro studies using a survivin inhibitor, terameprocol, led to a significant increase in apoptosis and decreased viability of tumor cells, thus presenting a potential therapeutic opportunity.

#### 2.2.2. AURKA

Aurora Kinase A (AURKA) is a serine/threonine kinase that contributes to the regulation of cell cycle progression. High level of AURKA mRNA expression was found in ENKTL patient samples and cell lines [19,20], along with protein detection by immunohistochemistry. AURKA is located at a frequently (> 50%) amplified locus in lymphomas derived from ENKTL [18,19]. The main cellular role of AURKA is in promoting mitosis [28], however it has recently been implicated to play a part in cell proliferation through *MYC* and *WNT* signaling, whilst inhibiting *TP53* [29,30,31]. Novel small molecule AURKA inhibitor (MK-8745) in NK-cell lines revealed a significant increase in apoptosis and cell cycle arrest suggesting a potential avenue for therapeutic targeting [19]. Furthermore, AURKA overexpression predicts resistance to taxane chemotherapy and AURKA inhibition has been shown to sensitize tumors to paclitaxel [28].

#### 2.2.3. C-MYC

C-myc, a transcriptional target of the EBV proteins, EBNA and LMP1, appears to play a considerable role in the transcriptional deregulation of ENKTL [32]. In addition to a potential role in global microRNA repression (see below), several Myc-induced transcriptional targets, such as EZH2 and RUNX3, have been reported to be upregulated in ENKTL. Although therapeutic strategies targeting MYC are being investigated in other lymphomas, they are yet to be explored in ENKTL [33].

#### 2.2.4. EZH2

Myc-induced downregulation of the microRNAs miR-26a and miR-101 leads to upregulation of their target Enhancer of Zeste Homolog 2 (EZH2) in ENKTL tumor tissue and cell lines [34]. EZH2 is a H3K27-specific histone methyltransferase and a component of the polycomb repressive complex 2 (PRC2), involved in the epigenetic maintenance of repressive chromatin marks [35]. Recent studies have shown that EZH2 can also function as a transcriptional co-activator via a non-canonical pathway, which has been shown to be the mechanism by which EZH2 promotes cell growth in ENKTL cells [36]. Phosphorylation of EZH2 by JAK3 mediates this switch from histone methyltransferase to transcriptional co-activator, resulting in the upregulation of a set of genes that are involved in DNA replication, cell cycle, biosynthesis, stemness and invasiveness [34,36]. Of note, a JAK3 inhibitor was able to significantly reduce the growth of ENKTL cells in vitro, thereby providing a potential pathway for the development of novel therapeutics. EZH2 inhibitors are being investigated in clinical trials for B-cell lymphomas, however their clinical efficacy is yet to be assessed in ENKTL [37].

#### 2.2.5. RUNX3

Runt-domain transcription factor 3 (RUNX3), a master transcriptional regulator in major developmental pathways, is another gene that is positively regulated by C-MYC [38]. RUNX3 can function as either a tumor suppressor or oncogene, depending on tumor type [39]. In cytotoxic T and NK cells, it has been shown to mediate transcriptional activation of genes involved in lymphocyte activation, proliferation, and effector function, which include interferon gamma (IFN-gamma) perforin and granzyme B [40]. RUNX3 expression is upregulated in ENKTL, as well as in the majority of high-grade B-cell lymphomas and peripheral T-cell lymphomas [38]. SiRNA -induced silencing of RUNX3 resulted in increased apoptosis and reduced cell proliferation in ENKTL cell lines, suggesting an oncogenic role for RUNX3 in ENKTL. Inhibition of MYC by a novel small molecule inhibitor, JQ1, resulted in downregulation of RUNX3 transcripts [38]. The precise mechanism of RUNX3-mediated reduction in cellular proliferation and increased apoptosis remains to be further delineated.

Among the deregulated single genes described above, EZH2, Survivin and AURKA have promising translational impact and may serve as potential therapeutic targets. In particular, JAK3 inhibition appears to be an attractive therapeutic approach and evaluation of JAK3 inhibitors as modulators of non-canonical EZH2 activity in clinical trials is warranted.

### 2.3. Deregulated Signaling Pathways

#### 2.3.1. JAK/STAT

Several studies have provided evidence supporting a role for the Janus Kinase/Signal Transducer and Activator of Transcription (JAK/STAT) pathway in ENKTL lymphomagenesis [41]. Gene expression profiling has shown that members of the JAK/STAT pathway are differentially expressed in ENKTL tumor cells compared to normal NK cells [17,42]. Whilst the frequency of JAK3 activating mutation in ENKTL appears to vary across studies in different ethnic populations (0–35%) [43,44,45,46], JAK3 phosphorylation is detected in 87% of the tested tumors and about 20% of cases is a result of activating mutations in the pseudo kinase domain of JAK3 [46], indicating that the constitutive activation of JAK3 pathway in ENKTL can arise from mechanisms other than mutation.

#### 2.3.2. PDGF pathway

Platelet derived growth factor receptor alpha (PDGFRα) is a receptor tyrosine kinase mediating important cell functions such as migration, proliferation, and cell survival and known to interact with PI3K/AKT and STAT signaling pathways [47]. PDGFRα gene and protein, including its phosphorylated form, is overexpressed in ENKTL, indicating activation of this pathway [17]. The tyrosine kinase inhibitor, imatinib mesylate, induced concentration-dependent growth inhibition in a PDGFRα-expressing ENKTL cell line, MEC04 [17]. The cause of PDGFRA deregulation in ENKTL remains uncertain and is not a result of genomic imbalances, gene mutations or overrepresentation of the H2α haplotype, the latter is known to result in up-regulation of PDGFRA in glioblastoma [17].

#### 2.3.3. NOTCH-1

The NOTCH signaling pathway is a highly conserved pathway with key roles in a wide variety of developmental processes and cancer. Studies have shown an enrichment of genes in the NOTCH signaling pathway in ENKTL [17,19]. Two NOTCH inhibitors, which are potent inhibitors of γ-secretase and Notch processing, induced significant growth inhibition in two NK-cell lines, providing preliminary evidence for the potential therapeutic targeting of this pathway [19]. However, these agents are yet to be evaluated in clinical trials.

#### 2.3.4. NF-κB

The relevance of the NF-κB pathway in ENKTL has been highlighted in two GEP studies, which found significant enrichment of genes in this pathway in ENKTL [17,20]. However, a separate study did not confirm the same findings [19]. Further research is therefore required to investigate the overall significance of NF-κB pathway in ENKTL. Although targeting NF-κB through bortezomib-based combinations has been attempted in ENKTL, data are only available on small cohorts of patients and larger trials are required for a more comprehensive assessment of efficacy and safety [48,49].

#### 2.3.5. Other Signaling Pathways

The VEGFR and AKT signaling pathways may also play a role in the pathogenesis of ENKTL [6,50]. The VEGFR inhibitor, bevacizumab, has been investigated in combination with cyclophosphamide, doxorubicin, vincristine, prednisolone (CHOP) in a small, heterogeneous group of patients with T-cell lymphomas [51]. The number of ENKTL patients was small and they were analysed together with PTCL patients. The overall response rate was 53%, suggesting a modest efficacy. ENKTL are known to have a poor response to anthracycline-based therapy, therefore clinical trials evaluating this agent in combination with L-asparaginase based regimens may be of value. AKT inhibitors are also under evaluation in clinical trials of unselected patients with relapsed lymphoma but their clinical efficacy in ENKTL remains to be established [52].

Among the pathways discussed, JAK/STAT and NF-κB are the best studied in ENKTL. The drugs targeting these pathways have made significant progress in terms of clinical development and have the greatest translational potential in the future.

## 3. Insights from Copy Number Analysis 

**Figure 1:**
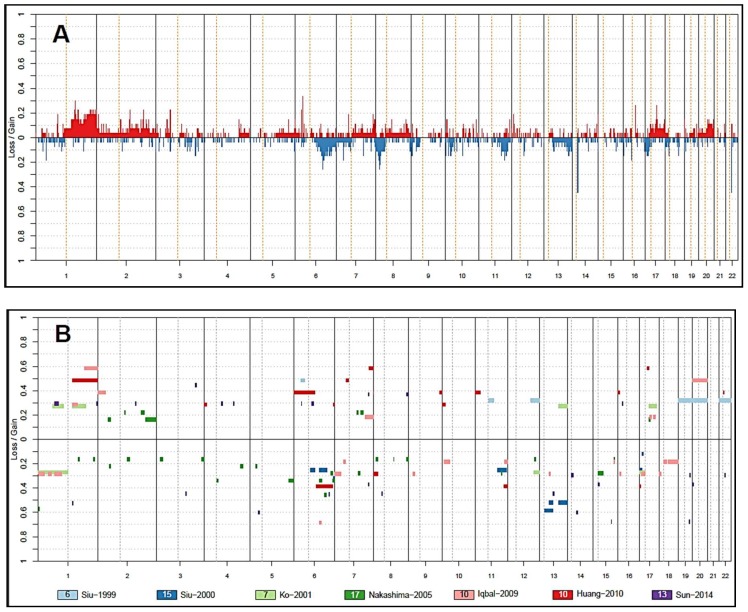
Penetrance plots of copy number aberrations (CNA) of ENKTL in comparison with published literature. Orange vertical line indicates centromere position. *Y*-axis indicates frequency of CNA. (**A**) Penetrance plots of 29 cases of ENKTL tested using Oncoscan molecular inversion probe assay [22]; (**B**) summary of recurrent CNA in previously published data. Recurrent CNA defined as aberrations occurring in two or more samples in each study (Table S1B). Number of samples in each study is indicated in the colour boxes.

Although no genetic abnormalities specific to ENKTL has been identified, studies based on comparative genomic hybridization (CGH) and loss of heterozygosity (LOH) analyses have identified recurrent gains and losses mapping to several chromosomal regions [17,18,53,54,55,56,57] (Figure 1 and Table S1), with one study finding up to 177 recurrent chromosomal gains and 35 losses [57].

### 3.1. Copy Number Loss (Potential Tumor Suppressor Genes)

The most consistently detected chromosomal abnormality among the studies to date is deletion of chromosome 6q, in particular 6q21-25. Sun et al. tried to define a minimal tumor suppressor gene-containing region involved in del6q25 through loss of heterozygosity (LOH) and homozygosity mapping of deletion analyses, followed by quantitative multiplex polymerase chain reaction analysis on 37 nasal and nasal-type NK/T-cell lymphoma patients using a panel of 25 microsatellite markers, covering the 6q21-q25 region and identified a 2.6 Mb interval located between TIAM2 and SNX9 genes [58]. Subsequent development of array CGH has allowed better resolution and identification of the specific region. The data from gene expression studies supporting a tumor suppressor role for several candidate genes identified within the commonly deleted 6q21 region are summarized below. Most of the available evidence is correlative and supplemented by preliminary in vitro functional studies in ENKTL cell lines.

#### 3.1.1. PRDM1

*PRDM1* encodes the transcription factor Blimp-1, which is a multi-functional master regulator of plasma cell differentiation [59], its role in ENKTL has been examined by several groups. Iqbal and colleagues found generally low expression of PRDM1 in ENKTL [18]. In addition to 6q21 deletion, they also detected mutations resulting in truncated forms of PRDM1. A further mechanism for the low expression of PRDM1 (presumably for 6q21 intact cases) is methylation of CpG islands 5′ of the *PRDM1* gene; highly methylated CpG islands 5′ of PRDM1 correlated with low expression of the transcripts, and reversal of methylation by Decitabine induced expression of PRDM1 with accompanying cell death [18,60]. Interestingly, another study found discrepant results in PRDM1 mRNA levels (which were under-expressed in cell lines but increased in a subset of primary tumors), independent of the presence or absence of 6q21 deletion [17]. Moreover, Ng et al. found overexpression of BLIMP1 protein by immunohistochemistry in ENKTL tumor tissue [32]. In view of these discrepancies, further studies are required to clarify the true significance of PRDM1 in the pathogenesis of ENKTL.

Interestingly, it has been shown that PRDM1 expression in ENKTL tumor samples may be of prognostic significance with positive expression being associated with longer progression free and overall survival [61]. In cases with negative staining of PRDM1, transcripts of PRDM1 were still detected, suggesting a possible post-transcriptional regulation at play, such as one mediated by miR-223 (see below).

#### 3.1.2. HACE1

HACE1 encodes a novel E3 ubiquitin ligase, which has been proposed as a tumor suppressor gene in multiple human cancers, including Wilm’s tumor [62]. HACE1−/− mice are spontaneously prone to developing multiple malignant tumors in various organs. It was found to be significantly downregulated in ENKTL through a combination of monoallelic 6q21 deletion and cytosine phosphate guanine (CpG) island methylation [17,63], but functional studies have yet to conclusively establish the role of HACE1 in the pathogenesis of ENKTL [64,65].

#### 3.1.3. PTPRK

PTPRK encodes receptor-type tyrosine-protein phosphatase κ, the only protein tyrosine phosphatase (PTPase) among the 3 PTPases located on chromosome 6q21 that interacts with STAT3. Chen et al. demonstrated that the loss of PTPRK expression due to monoallelic deletions and aberrant promoter hypermethylation likely contributes to lymphomagenesis by activating the STAT3 oncoprotein in ENKTL cells [66]. Furthermore, in their study of 27 patients with ENKTL, the authors found that underexpression of PTPRK protein in tumor cells correlated with advanced-stage disease, and that PTPRK promoter hypermethylation in tumors possibly correlated with a poorer prognosis in 17 patients treated with the steroid, methotrexate, iphosphamide, l-asparaginase, etoposide (SMILE) protocol [66]. As methylation of the PTPRK promoter can be reversed by demethylating agent, 5-aza-dC, epigenetic therapy may be able to target PTPRK-mediated cell signaling in ENKTL [66].

#### 3.1.4. Other Candidate Genes in the Commonly Deleted 6q21 Region

ATG5 is a protein involved in the autophagy pathway as well as apoptosis [67]. Low expression of ATG5 in ENKTL was found in two studies [17,18]. However, the role of ATG5 in the pathogenesis of ENKTL remains controversial given its role in tumorigenesis and tumor suppression in other malignancies [40]. AIM1 is an actin-binding protein that suppresses cell migration [68] and is underexpressed in ENKTL [17,18]. Similar to PRDM1, highly methylated CpG islands 5′ of AIM1 correlated with low expression of the transcripts [18]. FOXO3 is a member of the forkhead family of proteins. Re-expression of FOXO3 suppressed the proliferation of an NK-cell line with haploinsufficiency as the suggested mechanism responsible for FOXO3 inactivation in ENKTL [69]. Further work is however required to evaluate the functional role of this protein in ENKTL.

In summary, PRDM1 remains the best evaluated putative tumor suppressor gene in ENKTL. Nonetheless, restoring the function of an inactivated tumor suppressor gene remains more challenging than inhibiting a hyperactivated oncogene. Hence, development of clinically useful strategies in this field remains a work in progress. Epigenetic silencing of PRDM1 and PTPRK using hypomethylating agents, however, may be an attractive therapeutic option for ENTKL.

### 3.2. Other Chromosomal Gains and Losses

Other less well-characterized genomic alterations include gains at chromosome 1q, 2q, 6p, 7q, 17q, and 20q, and losses at 11q, 13q and 17p. Gene expression analyses showed that among the gains, the majority of genes showing more than two-fold-overexpression play a role in cell proliferation, cell cycle progression and metabolic processes. There was downregulation of major functional groups of genes within the most frequently lost regions, including genes involved in transcriptional repression (*NCOR1*, *PRDM1a* and *ZNF10*), and tumor suppressors (*FOX01A*, *CHFR*, *CDKN2C* and *MAP2K4*) [17,18]. Negative regulators of the cell cycle and cytoskeletal organization (*KIF2C*, *MCM10* and *MAD2L2*) and pro-apoptotic genes, such as *P53AIP1*, *PRKACB*, *DFFA* and *SH3GLB1*, were also downregulated [17,18].

Two findings stand out particularly among the chromosomal abnormalities found outside the 6q21-25 region in terms of their clinical relevance. Sun et al. found that six out of thirteen patients with ENKTL had a previously unreported recurrent loss of 8p11.23, which correlated with a significantly worse prognosis compared to patients without this loss [57]. Although the authors postulate *ADAM3A* as a candidate gene at this locus, its definitive role has yet to be established.

In a recent study of 12 EBV-positive PTCL and 29 ENKTL using the Oncoscan-molecular inversion probe array, Ng et al. highlighted that loss of 14q11.2 is present in the majority of EBV-positive PTCL and in a subset of ENKTL. Loss of 14q11.2 correlates with T-cell origin in their study samples and may be a potentially useful marker of T-cell lineage [22].

## 4. Insights from Genome Wide Association Studies

ENKTL is prevalent among Asians and the indigenous populations of Mexico, Central and South America, suggesting that the risk of developing the disease depends on both immunogenetic background and environmental factors [70]. However, the molecular and genetic mechanisms underpinning individual susceptibility are poorly understood. Thus far, there have been two major studies investigating the genetic risk of ENKTL.

Based on previous observations that several cytotoxic T-lymphocyte (CTL) defined epitopes were mapped to EBV latent membrane proteins (LMPs) restricted with HLA-A2, -A11 or -A24 antigens, Kanno and colleagues performed a HLA-A allotyping study in 25 patients with ENKTL compared with 303 healthy controls of Japanese descent and reported significantly lower frequency of the HLA-A*0201 allele in ENKTL [71]. The authors therefore suggested that the HLA-A*0201-restricted CTL responses to LMPs in EBV-infected pre-neoplastic and/or neoplastic cells of NK-cell lineage may function in vivo to suppress the development of overt lymphoma, raising the possibility of the development of immune therapy.

A subsequent first genome-wide association study (GWAS) of 189 extranodal ENKTL and 957 controls of Chinese descent by Li et al. did not reveal a similar finding of lower frequency of HLA-A*0201 in their study population [72]. Instead, the authors showed that the single nucleotide polymorphism (SNP) rs9277378 (located in HLA-DPB1) had the strongest association with ENKTL (*p* = 4.21 × 10^−19^). HLA-DPB1 is the β1 subunit of the HLA-DP heterodimer involved in extracellular antigen presentation to CD4-positive T-cell lymphocytes [73]. This finding thereby highlights the importance of HLA-DP antigen presentation in the pathogenesis of ENKTL. The study did not find an association between HLA-DPB1 and other EBV-related malignancies, such as nasopharyngeal carcinoma in southern Chinese populations and Hodgkin’s lymphoma in Europeans from publicly available GWAS results, suggesting that these diseases might have distinct molecular mechanisms of pathogenesis beyond EBV infection.

Interestingly, Li and colleagues also found that, in the discovery cohort, the association between the HLA-DPB1 rs9277378*A risk allele and increased risk of ENKTL was much stronger in patients without hepatitis B virus (HBV) infection, compared with patients who had concurrent HBV infection. As the HLA-DPB1 rs9277535*A risk allele was previously reported to be associated with improved clearance of HBV [74], the authors postulate that individuals with greater HBV clearance ability would have a higher risk of ENKTL. Indeed, in a recent case control study including 417 ENKTL cases and 488 age- and sex-matched controls, individuals who were naturally immune to HBV were significantly more likely to be diagnosed with ENKTL (*p* = 0.001) [75].

These findings all suggest that SNPs associated with host immunity and inflammatory responses, including to HBV, play an important role in the individual susceptibility and subsequent development of ENKTL. Determining the precise mechanisms underlying these associations could be relevant to the development of personalized and successful immunotherapies.

## 5. Insights from Mutational Profiling 

**Figure 2:**
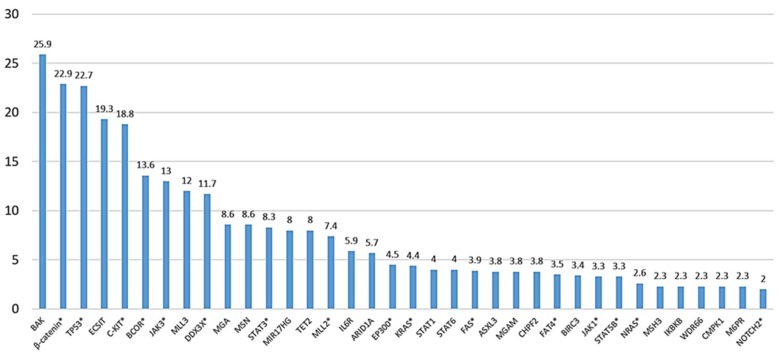
Overall frequency of somatic gene mutations identified in ENKTL. *Y*-axis indicates percentages. Overall frequency calculated based on total number of cases reported in each study (refer Table S2 for details). Mutations identified in two or more studies are highlighted with *.

Several studies have focused on elucidating the mutational landscape of ENKTL (Figure 2, Table 1, and Table S2). The genes are related to the various important pathways mentioned above, including JAK-STAT-pathway members, epigenetic modifiers, nucleoside binding, biological adhesion and plasma membrane, RNA helicases and tumor suppressors, indicating that abnormalities of these biological functions and processes may have substantial roles in tumor pathogenesis. NK- and T-cell derived ENKTL as defined by TCR gene rearrangement assays showed no significant differences in their mutational landscape [76].

### 5.1. JAK/STAT Pathway Associated Genes

JAK3, STAT3 and STAT5B mutations leading to constitutive activation of the JAK/STAT pathway have been found in up to 35% of cases of ENKTL [42,44,46]. A few studies have found none or a much lower frequency of JAK3 mutations and more frequent JAK3 phosphorylation instead [43,45,76,77], suggesting that alteration of JAK3 function is predominantly attributed to phosphorylation rather than mutation and the variable frequency of occurrence reported may be due to the different detection platforms or a reflection of population/ethnicity-related differences. Similarly, STAT3- and STAT5B-activating mutations are found in only 6% of cases each [78], while STAT3 phosphorylation activation at Tyr705 is found in about 90% of cases [17,41]. It has been postulated that since PTPRK normally dephosphorylates phospho-STAT3, under-expression of PTPRK, whose encoding gene is located in the commonly deleted 6q region, due to deletion or promoter hypermethylation may lead to STAT3 activation [66].

In their study, Sim et al. found two novel JAK3H583Y and JAK3G589D somatic mutations which the authors suggested had more oncogenic potential and were also more sensitive to Tofacitinib, a potent inhibitor of JAK1 and JAK3 [77]. The same authors also found that although the frequency of STAT3 mutations was low in patients with ENKTL, STAT3-mutant ENKTL cells were sensitive to the STAT3 inhibitor, Stattic, but not to Tofacitinib. A selective inhibitor of JAK3 (PRN371) has recently been demonstrated to have more potent anti-tumor activity than Tofacitinib in a xenograft model of ENKTL with a JAK3 mutation [79]. These results indicate that JAK3 and STAT3 are candidate therapeutic targets in patients with ENKTL. Phase II clinical trials of Ruxolitinib, a JAK1/2 inhibitor approved for myelofibrosis, are now in progress for patients with relapsed ENKTL [80].

### 5.2. Epigenetic Regulators

BCOR and MLL2 are epigenetic regulators known to be mutated in other malignancies. BCOR interacts with some histone-deacetylase-family genes and MLL2 encodes a histone methyltransferase [81,82]. BCOR and MLL2 mutations are also seen in ENKTL, with apparent mutual exclusivity [42,76,83]. While the clinical and biological relevance of these mutations remains unclear, epigenetic modification may play a role in tumor pathogenesis as both genes are involved in chromatin modification. Mutations in other epigenetic modifiers ASXL3, ARID1A and EP300 have also been found at varying frequency [76].

### 5.3. DDX3X

DDX3X is a RNA helicase gene and mutants exhibit decreased RNA-unwinding activity, loss of suppressive effects on cell-cycle progression in NK cells and transcriptional activation of NF-KB and MAPK pathways [76]. DDX3X mutations are frequent and reported in up to 50% of ENKTL cases [76,83]. Jiang et al. found that mutations of DDX3X seldom overlap with TP53, suggesting that these two genes may be involved in very closely related biological processes [76].

### 5.4. TP53 and Pro-Apoptotic Genes

TP53 is a crucial tumor suppressor gene known to be mutated in several malignancies [84]. TP53 mutations are present in up to 63% of cases of ENKTL [42,76,85,86,87,88]. They have been associated with advanced stage disease, suggesting that TP53 mutation represents a secondary rather than an initiating oncogenic event in ENKTL [89]. Indeed, Jiang et al. demonstrated that individuals with mutations in DDX3X and TP53 had much worse clinical prognosis than individuals without mutations in these two genes [76]. Nevertheless, TP53 is found under-expressed in ENKTL transcriptomic analyses, and the observation that EBNA1 promotes TP53 degradation has to be taken into account in the consideration of TP53 pathway as a possible target for ENKTL therapy [50]. Failure of apoptosis in ENTKL due to TP53 mutations may be enhanced by mutations in apoptotic protein FAS [90]. The precise mechanisms involved require further evaluation.

### 5.5. ECSIT

ECSIT has been identified as a cytoplasmic protein involved in Toll-like receptor (TLR), transforming growth factor beta/bone morphogenetic protein (TGF-β/BMP) signaling pathways, and has been shown to play an important role in TLR4-mediated signals to activate NF-κB [91]. In line with published data, the ECSIT-T419C mutation activates the NF-κB pathway in ENKTL and induces secretion of proinflammatory cytokines. Wen and colleagues recently identified a hotspot mutation encoding ECSIT-V140A in 19.3% of 88 ENTKL cases. This mutation was associated with younger age, advanced clinical stage, high International Prognostic Index (IPI), splenomegaly, presence of hemophagocytic syndrome (HPS) and poor prognosis [92]. Preliminary data on two ENKTL patients expressing ECSIT-V140A revealed that a combination of thalidomide and dexamethasone was effective in reversing the HPS. Additional studies are necessary to determine the frequency and role of the ECSIT-V140A mutation and the efficacy of the proposed regimen in ENKTL patients expressing this mutation.

### 5.6. Pro-Survival Signaling Pathways

The KIT proto-oncogene encodes a receptor tyrosine kinase which plays an important role in normal hematopoiesis, gametogenesis and melanogenesis. KIT mutations are well-known in the oncogenesis of gastrointestinal stromal tumors and mastocytosis, and tyrosine kinase inhibitors have proven useful in the treatment of these diseases [93]. KIT mutation is reported in up to 52% of ENKTL cases but a gain of function as a result of the mutations has not been shown [94]. Mutations in RAS family genes (KRAS, NRAS) [42,76,83,86,95], as well as the beta-catenin pathway (CTNNB1) have also been described in ENTKL [86,95].

## 6. Epigenetic Dysregulation in ENKTL

### 6.1. Dysregulated Promoter Methylation 

The epigenetic mechanisms underpinning genetic dysregulation in ENKTL are beginning to be understood. On a global level, there appears to be widespread promoter hypermethylation in most ENKTL compared to normal NK cells, assessed in a study using methyl-sensitive cut counting and reduced representation bisulfite sequencing on 12 ENKTL cases and seven NK cell lines [96]. Promoter hypermethylation was especially prominent at “poised genes”, which are genes showing histone marks associated with both activated and repressed states. Several known tumor suppressor genes, including *BCL2L11* (*BIM*), *DAPK1*, *PTPN6* (*SHP1*), *TET2*, *SOCS6*, and *ASNS* show reduced gene expression correlating with promoter hypermethylation in NK cell lines [96]. Ectopic expression of BIM and SOCS6 in non-expressing NK cell lines resulted in growth inhibition and sensitization to chemotherapy-induced apoptosis for BIM, whilst lack of ASNS expression in NK cell lines was associated with increased sensitivity to L-asparaginase treatment. These findings suggest a potential role for methylation markers in guiding therapeutic strategies in ENKTL [96].

Methylation of other putative tumor suppressor genes was also found in an earlier study by Siu LL et al. (2002), in which the promoter methylation status of five putative tumor suppressor genes (*p15*, *p16*, *p73*, *hMLH1* and *RARβ*) was assessed by methylation-specific polymerase chain reaction (MSP) in 33 patients samples using a candidate gene approach [56]. The *p73* gene in particular, was methylated in a large majority (94%) of the cases, and its demethylation with 5-azacytidine resulted in re-induction of gene expression in vitro. Other methylated genes included *hMLH1* (63%), *p16* (63%), *p15* (48%), and *RAR*β (47%). Interestingly, MSP identified two cases of occult marrow involvement and detected tumor involvement in a histologically negative oropharyngeal biopsy, suggesting methylation testing to be of potential diagnostic utility.

Other tumor suppressor genes with methylated promoters have been described above, including *PRDM1* [18], *AIM1* [18], *HACE* [17,63] and *miR-146a* [97].

### 6.2. MicroRNA deregulation in ENKTL

In the past two decades, microRNAs, a class of short, ~22 nucleotide-long non-coding RNAs have been shown to mediate an additional layer of regulatory complexity in gene expression [98]. Most microRNAs negatively regulate the expression their target genes. The role of microRNAs in ENKTL have been recently reviewed [99,100,101]. On a global level, miRNAs in ENKTL are predominantly down regulated compared to normal NK cells and they include miR-150, miR-101, miR-26a, miR-26b, miR-28-5, miR-363, and miR-146 [32,102]. Upregulated predicted targets are enriched for genes involved in cell-cycle related, p53 and MAPK signaling pathways. Several of the predicted targets, including *MUM1*, *BLIMP1*, and *STMN1* were verified by immunohistochemistry to be overexpressed in ENKTL tumors [32]. On the other hand, a few microRNAs, such as mir-21 and miR-155 have been shown to be overexpressed in ENKTL, with pro-oncogenic consequences [32,103]. Some microRNAs have also been shown to be of prognostic significance. Low expression of miR-146a in ENKTL was associated with a poorer prognosis [97]. Another study reported that elevated plasma levels of miR-221 in ENKTL patients was associated with shorter overall survival and may serve as an adverse prognostic marker [104]. The evidence supporting a role for specific microRNAs in ENKTL lymphomagenesis is summarized in Table 2.

### 6.3. Mechanisms of microRNA Dysregulation in ENKTL

The mechanisms underlying the widespread downregulation of miRNAs in ENKTL are beginning to be delineated. MYC, a key transcriptional regulator known to cause extensive repression of miRNA, has been shown to be overexpressed in ENKTL and may thus reprise its global miRNA repressor role in the setting of ENTKL [32,108]. EBV may play a role in the deregulation of miRNAs since downregulation of let-7g, let-7a, and let-7c, and up-regulation of miR-155 in ENKTL have been demonstrated in other studies to be regulated by EBV [109,110]. In addition, epigenetic deregulation due to promoter methylation has been reported to result in the deregulation of miRNAs in ENKTL, such as miR-146a and miR-124-1 [97,111].

## 7. Potential Immunotherapeutic Targets in ENKTL

Programmed cell death-1 (PD-1) inhibition has revolutionized immunotherapy in cancer in the recent past [112]. We have previously performed GEP on 29 ENKTL using FFPE tissues and derived the differentially expressed genes between tumor samples and control tissues [22] (GEO database GSE90597). We observed upregulation of PD-Ligand 1 (PD-L1, also known as CD274) mRNA in tumor compared to control tissues (Table S3). Indeed, PD-L1 expression on ENKTL has been demonstrated by immunohistochemistry [113] while in vitro studies using ENKTL cell lines have also shown that overexpression of LMP1 results in upregulated PD-L1 expression [114]. Interestingly, STAT-3 mutations have been proposed as a mechanism by which ENKTL cells upregulate PD-L1 [115]. Taken together, these data support the evaluation of PD-1 checkpoint inhibitors in the clinical setting. Kwong et al. described a case series of seven patients with relapsed ENKTL, all of whom responded to treatment with pembrolizumab, an antibody against PD1 [116]. Of note, two patients achieved a complete response in all parameters (clinical, radiologic, morphologic and molecular) assessed. PD-L1 expression data were available in five cases, four of which showed uniform strong expression and one showed weaker expression in ~20% of the cells. The response to pembrolizumab, however, did not show a clear correlation with PD-L1 expression in tumor cells. Therefore, new biomarkers that can predict clinical response to checkpoint inhibition are needed [117].

A better understanding of the differential expression of PD-L1 and PD-1 on malignant versus immune cells in the microenvironment maybe a crucial step in the process of understanding responses to check point inhibitors. Most studies reporting upregulation of PD-L1 relied on single marker immunohistochemistry. Although visual scoring of staining intensity is of clinical value in the hands of an experienced hematopathologist, the data generated remain subjective with limited reproducibility [118]. Furthermore, there are several commercial clones of PD-L1 antibodies with different sensitivity and specificity. Currently, there remains a lack of standardization with regards to the staining protocol and quantification method with consequent variability in the data reported in the literature [119].

Adoptive immunotherapy using antigen-specific T cells targeting EBV-associated viral antigens has been employed in the treatment of ENKTL, with one study showing complete response in four out of six patients with active disease, and continued complete response in five of five patients in a first or later remission cohort [120]. In these patients, the therapy appeared to be well-tolerated, with 10 of 11 patients showing no toxicity attributed to the therapy, and one with a possible inflammatory response [120].

CD38 is a pleiotropic glycoprotein belonging to a complex family of enzymes of the cell surface and involved in the catabolism of extracellular nucleotides [121]. Our GEP data have demonstrated upregulation of CD38 gene in ENKTL tumor compared to control tissues (Table S3, GEO database GSE90597). CD38 protein is strongly expressed in 50% of ENKTL and this is associated with significantly inferior outcomes compared to those with weak expression, indicating the potential role of CD38 as a therapy target [122]. The humanized monoclonal antibody, daratumumab, which targets CD38 has been approved for the treatment of relapsed multiple myeloma and is being explored in other lymphoproliferative disorders [123]. Recent in vitro studies revealed that daratumumab has good efficacy against ENKTL [124]. This was highlighted in the clinical setting by the dramatic response of a patient with relapsed refractory ENKTL to daratumumab monotherapy [125]. While these data suggest that CD38 is an attractive therapeutic target in ENKTL, most of the research on daratumumab has been in multiple myeloma and ENKTL-specific mechanisms of action and resistance will have to be investigated.

## 8. Opportunities for Translation

### 8.1. Refining Diagnosis

The novel data gleaned from genome-wide high throughput techniques have greatly improved our understanding of the molecular biology of ENKTL and has significant impact on disease diagnosis and classification. These techniques have allowed the identification of the gene signature of ENKTL, the unique set of genes that defines the genomic characteristics of the tumor and distinguishes it from other PTCLs [17,19,21]. It has also shown that lymphomas originating from NK cells are molecularly related to those derived from a subset of γδ T-cells that are characterized by a non-hepatosplenic presentation. Recent studies have further demonstrated that ENKTL is distinct from other overlapping and closely related diseases, such as EBV-positive T/NK-cell lymphomas presenting with nodal disease as well as those presenting in children and young adults [22,26]. While the data have led to an increased understanding of the tumor biology, the translation of the genomic data into clinically applicable diagnostic biomarkers remains challenging and confounded by the rarity of the disease and limited tissue availability.

### 8.2. Risk Stratification

Current risk stratification models of ENKTL utilize exclusively clinical criteria [126]. Recent studies have identified several molecular markers of potential prognostic significance in ENKTL. These include cytogenetic data, such as loss of 8p11.23, which correlated with a significantly worse prognosis in patients with ENKTL [57], mutational status of genes such as DDX3X and TP53 [76], and promoter methylation status of genes such as PTPRK [66]. In addition, tissue-based expression markers of potential prognostic significance include BLIMP1 [61] and miR-146a [97]. A circulating marker, miR-221, was also associated with a less favorable long-term outcome in ENKTL patients [104]. Whilst there is an emerging body of evidence to support the utility each of these markers, a direct comparison of their effectiveness against currently used clinical indices remains to be investigated. The early identification of patients who will not respond to standard therapy is important for the selection of these individuals for clinical trials with novel agents.

### 8.3. Conclusions, Promising Therapeutic Targets and Future Directions

**Figure 3:**
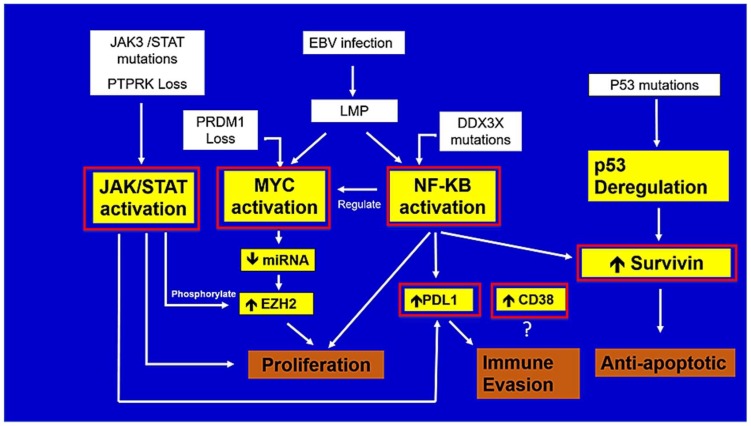
Proposed model of pathogenesis of ENKTL and potential therapeutic targets highlighted in red. EBV infection may provide a proliferative signal via MYC and NF-kB activation. JAK/STAT activation contributes through its known pro-proliferative functions and also via phosphorylation of EZH2 resulting in non-canonical activation of proliferative pathways. Anti-apoptotic effects may be driven by survivin as a result of p53 deregulation and NF-kB activation. Finally, PD-L1 plays a crucial role in immune evasion. The role of CD38 in the molecular biology of ENKTL is still under investigation.

Integration of massive sequencing strategies and gene expression has characterized the driver genetic alterations in ENKTL, as in other PTCLs [16]. These studies have identified oncogenic pathways that can be potentially targeted by specific therapies (Figure 3). While major advances in our understanding of the biology of ENKTL have been made in the last decade, the diagnosis and classification of ENKTL is likely to be further refined by GEP and other genomic techniques in the near future. The identification of potential tumor suppressor genes, PRDM1 and PTPRK, and pro-survival signaling via MYC and NF-KB are of particular importance as key events in ENKTL pathogenesis (Figure 3 and Table 3). Similarly, the discovery of transcriptional dysregulation through RUNX3 and non-canonical functions of EZH2 in ENKTL have shed new light on the molecular biology of this disease. Despite the biological importance of these findings, their clinical relevance remains to be established. From a therapeutic translational standpoint, we propose that immune checkpoint inhibition is one of the most promising avenues for drug development in ENKTL (Figure 3 and Table 3) [127]. Further work is necessary to establish the cell surface expression patterns of PD-1 and PD-L1 and a better understanding of the intracellular regulation of PD-L1 expression in this disease. The discovery of CMTM6 as a novel regulator of PD-L1 through endosomal recycling would be an interesting area for further studies in ENTKL [128]. Importantly, clinical trials evaluating check point inhibition for ENKTL in both relapsed and up front settings are required. Clinical trials evaluating JAK inhibition as a therapeutic strategy in ENKTL are ongoing, supported by the strong preclinical evidence of the dysregulation of this pathway. Finally, we propose that CD38 may be an attractive target for future clinical trials based on its strong and uniform expression in ENKTL along with promising early clinical data. It would be of great interest to assess the combination of anti-CD38 antibodies or JAK inhibitors in combination with L-asparaginase based chemotherapy in the up front or relapsed setting.

## Figures and Tables

**Table 1:** Summary of the frequency of recurrent mutations in ENKTL. The overall frequency of mutations is expressed as a percentage of mutated cases out of the total number of cases tested. The reported frequency is available for comparison. Mutations reported in two or more studies are highlighted in bold. See Table S2 for further details.

Gene	Reported Frequency	No. of Positive Cases (Total No. of Cases Tested)	Overall Frequency (%)
*ARID1A*	6	6 (105)	5.7
*ASXL3*	4	4 (105)	3.8
***BCOR***	6–32	20 (147)	13.6
***b-catenin***	16–30	41 (179)	22.9
*BIRC3*	3	3 (88)	3.4
*BAK*	25.9	7 (27)	25.9
*CHPF2*	4	4 (105)	3.8
***C-KIT***	5–52	30 (170)	18.8
*CMPK1*	2	2 (88)	2.3
***DDX3X***	8–50	33 (283)	11.7
*ECSIT*	19	17 (88)	19.3
***EP300***	4–6	7 (156)	4.5
***FAT4***	2–8	4 (113)	3.5
***FAS***	4	3 (76)	3.9
*IKBKB*	2	2 (88)	2.3
*IL6R*	6	2(34)	5.9
***JAK1***	2-8	3 (90)	3.3
***JAK3***	5-35	36 (227)	13
***KRAS***	3–25	11 (251)	4.4
***MLL2***	2–18	14(190)	7.4
*MLL3*	12	3 (25)	12
*MSH3*	2	2 (88)	2.3
*M6PR*	2	2 (88)	2.3
*MIR17HG*	8	2 (25)	8
*MSN*	9	9 (105)	8.6
*MGAM*	4	4 (105)	3.8
*MGA*	9	9 (105)	8.6
***NRAS***	2–25	6 (227)	2.6
***NOTCH2***	2–25	3 (153)	2
*STAT1*	4	1 (25)	4
***STAT3***	1–26	30 (387)	8.3
***STAT5B***	2–6	8 (244)	3.3
*STAT6*	4	1 (25)	4
***TP53***	4–63	139 (611)	22.7
*TET2*	8	2 (25)	8
*WDR66*	2	2 (88)	2.3

**Table 2:** Micro RNA deregulation in ENKTL. This table summarizes the mechanisms by which miRNA deregulation leads to lymphomagenesis in ENKTL.

MicroRNA	Evidence Supporting Their Biological Significance	References
**MicroRNAs underexpressed in ENKTL**		
miR-146a	Overexpression of mir-146a suppressed cell proliferation, induced apoptosis, and enhanced chemosensitivity by inhibiting the NF-kB pathway via targeted downregulation of TRAF6. ENKTL patients with low miRNA-146a expression had higher frequency of non-response to chemotherapy.	Paik et al. 2011 [97]
miR-150	MiR-150 is expressed at lower levels in both ENKTL cell lines and tumor tissue compared to normal NK cells. Its aberrant downregulation induced continuous activation of the PI3K–AKT pathway, leading to telomerase activation and immortalization of cancer cells.	Watanabe et al. 2011 [105]
miR-26 and miR-101	Downregulation of miR-26a and miR-101 resulted in upregulation of their target Enhancer of Zeste Homolog 2 (EZH2) in ENKTL tumor tissue and cell lines.	Yan et al. 2013 [34]
miR-223	MiR-223 targets PRDM1, a potential tumor suppressor gene in ENKTL: (i) miR-223 and PRDM1 exhibited inverse patterns of expression in ENKTL tissues and cell lines; (ii) PRDM1 was identified as a direct target gene of miR-223 by luciferase assays; (iii) ectopic expression of miR-223 led to downregulation of the PRDM1 protein in vitro whereas a decrease in miR-223 restored the level of PRDM1 protein.	Liang et al. 2014 [61]
miR-142-3p and miR-205	miR-142-3p and miR-205 are downregulated in ENKTL compared with normal thymic tissue. Mir-142-3p targets the proinflammatory cytokine interleukin 1 alpha (IL1A) and mir-205 targets the oncogene BCL6 in vitro.	Motsch et al. 2012 [102]
miR-10, miR-342-3p	Expression of miR-10a and miR-342-3p, which are downregulated in ENTKL tissues, is inversely correlated with protein expression of their predicted target gene, T-lymphoma invasion and metastasis inducing factor 1 (TIAM1).	Huang et al. 2016 [106]
**MicroRNAs overexpressed in ENKTL**		
miR-155 and miR-21	MiR-21 and miR-155 are over-expressed in ENKTL samples and cell lines. Mir-21 downregulates phosphatase and tensin homolog (PTEN) and programmed cell death 4 (PDCD4), whilst mir-155 directly targets SHIP1 in ENTKL cell lines. Both PTEN and SHIP1 are involved in the AKT signaling pathway.	Yamanaka et al. 2009 [103]
**EBV-encoded microRNAs**		
BART9	BART9 shows a pro-proliferative effect in two ENKTL cell lines (SNK6 and SNT16) that is mediated, at least in part by upregulation of LMP-1 levels.	Ramakrishnan etal. 2011 [107]

**Table 3:** Therapeutic targets with the greatest translational potential in ENKTL.

Therapeutic Targets or Signaling Pathway	Clinical Significance for Therapeutics	Reference
JAK-3	JAK-3 inhibition is shown to have potent anti-tumor activity in pre-clinical models. Clinical trials evaluating JAK inhibitors in ENKTL are in progress.	Sim et al. 2017 [77] Narisimagi et al. 2017 [79]
STAT-3	STAT-3 mutant ENKTL are sensitive to STAT-3 inhibition in vitro.	Sim et al. 2017 [77]
NF-kB	NF-kB upregulation is an important event in ENKTL pathogenesis. Bortezomib is being evaluated in early phase clinical trials.	Tang et al. 2016 [49]
CD38	CD38 is upregulated in ENKTL. Daratumumab has good in vitro efficacy and one case report documenting complete response.	Mustafa et al. 2017 [124] Hari et al. 2016 [125]
PD-1	PD-L1 is upregulated in ENKTL. Early clinical trials show potent single agent activity of anti PD-1 therapy in relapsed, refractory ENKTL.	Kwong et al. 2017 [116]

## Supplementary Materials

Supplementary materials can be found at http://www.mdpi.com/1422-0067/19/7/1931/s1.
