# Supplementary material for: The Genomics and Molecular Biology of Natural Killer/T-Cell Lymphoma: Opportunities for Translation
Source: Int J Mol Sci. 2018 Jun 30;19(7):1931. doi: 10.3390/ijms19071931 (PMC6073933; doi:10.3390/ijms19071931)
Supplement: Supplementary file 1 [file ijms-19-01931-s001.zip › Supplementary Tables and methodv2.docx]

Supplementary Tables

**Table S1A Details of studies reporting copy number aberrations in NK/T cell lymphomas**

| **No.** | **Study** | **Total no. of cases** | **No. of ENKTL cases** | **Method** |
| --- | --- | --- | --- | --- |
| 1 | Siu et al.  (1999)[[1](#_ENREF_1)] | 10: 5 ANKL, 5 ENKTL | 5 (6 samples)* | conventional CGH |
| 2 | Siu et al.  (2000)[[2](#_ENREF_2)] | 18 patients: 3 ANKL, 15 ENKTL | 15 (16 samples)** | LOH on 6q, 11q, 13q, 17p^^^ |
| 3 | Ko et al.  (2001)[[3](#_ENREF_3)] | 7: ENKTL | 7 | conventional CGH LOH of 6q, 1p and 17p^^^ |
| 4 | Nakashima et al.  (2005)[[4](#_ENREF_4)] | 27 patients:  10 ANKL, 17 ENKTL | 17 | aCGH |
| 5 | Iqbal et al.  (2009)[[5](#_ENREF_5)] | 8 cell lines: 3 ANKL, 4 ENKL, 1 severe CAEBV  7 clinical cases: 6 ENKTL, 1 ANKL | 10 ^ | aCGH |
| 6 | Huang et al.  (2010)[[6](#_ENREF_6)] | 8 ENKTL, 2 cell lines | 10 ^^ | aCGH |
| 7 | Sun et al.  (2014)[[7](#_ENREF_7)] | 13: ENKTL | 13 | aCGH |
| 8 | Ng et al.  (2017)[[8](#_ENREF_8)] | 12 PTCL EBV+, 29 ENKTL | 29 | OS-MIP array |

**includes 2 relapses, **includes 6 relapse, ^includes 4 cell lines, ^^includes 2 cell line, ^^^percentages calculated excludes noninformative / failed cases, ANKL; aggressive NK cell leukemia, ENKTL; extranodal NK/T cell lymphoma, nasal type, CAEBV; chronic active EBV infection, PTCL; peripheral T cell lymphoma NOS, CGH: comparative genome hybridization; LOH: loss of heterozygosity; aCGH: array comparative genome hybridization; OS-MIP: OncoScan Molecular Inversion Probe*

**Table S1B Number and frequency of copy number aberrations reported in ENKTL**

|  | Siu et al.  (1999)  [[1](#_ENREF_1)] | Siu et al.  (2000)  [[2](#_ENREF_2)] | Ko et al.  (2001)  [[3](#_ENREF_3)] | Nakashima et al.  (2005)  [[4](#_ENREF_4)] | Iqbal et al.  (2009)[[5](#_ENREF_5)] | Huang et al.  (2010)  [[6](#_ENREF_6)] | Sun et al.  (2014)  [[7](#_ENREF_7)] |  |
| --- | --- | --- | --- | --- | --- | --- | --- | --- |
| Chromosomal location |  |  |  |  |  |  |  |  |
| *Recurring gains* |  |  |  |  |  |  |  |  |
| 1p21-p31 |  |  | 2 (29) |  |  |  |  |  |
| 1p31.1 |  |  |  |  |  |  | 4 (31) |  |
| 1p32-pter | 1 (17) |  |  |  |  |  |  |  |
| 1p33-pter | 1 (17) |  |  |  |  |  |  |  |
| 1pter-q32 | 1 (17) |  |  |  |  |  |  |  |
| 1q21-q31 |  |  | 2 (29) |  |  |  |  |  |
| 1q21.1-q23.3 |  |  |  |  | 3 (30) |  |  |  |
| 1q21-q44 |  |  |  |  |  | 5 (50) |  |  |
| 1q22-q25.3 |  |  |  |  |  |  |  |  |
| 1q23-q31 | 1 (17) |  |  |  |  |  |  |  |
| 1q25.3-q44 |  |  |  |  |  |  |  |  |
| 1q31.3-qter |  |  |  |  | 6 (60) |  |  |  |
| 1q44 |  |  |  |  |  |  | 4 (31) |  |
| 2p11-p16 | 1 (17) |  |  |  |  |  |  |  |
| 2p16.3-p21 |  |  |  | 3 (17) |  |  |  |  |
| 2p23.1-pter |  |  |  |  | 4 (40) |  |  |  |
| 2q |  |  | 1 (14) |  |  |  |  |  |
| 2q12-q14 |  |  | 1 (14) |  |  |  |  |  |
| 2q13 |  |  |  | 4 (24) |  |  |  |  |
| 2q22-q32 |  |  | 1 (14) |  |  |  |  |  |
| 2q22.2-22.3 |  |  |  |  |  |  |  |  |
| 2q24.1 |  |  |  |  |  |  | 4 (31) |  |
| 2q24.2 |  |  |  |  |  |  |  |  |
| 2q31-q32 |  |  | 1 (14) |  |  |  |  |  |
| 2q31.3-32.2 |  |  |  | 4 (24) |  |  |  |  |
| 2q32.3-q33.1 |  |  |  |  |  |  |  |  |
| 2q33.1 |  |  |  |  |  |  |  |  |
| 2q33-q36 |  |  | 1 (14) |  |  |  |  |  |
| 2q33.1-q37.3 |  |  |  | 3 (17) |  |  |  |  |
| 3p21 | 1 (17) |  |  |  |  |  |  |  |
| 3q |  |  | 1 (14) |  |  |  |  |  |
| 3q26.1 |  |  |  |  |  |  | 6 (46) |  |
| 4p15-q25 | 1 (17) |  |  |  |  |  |  |  |
| 4p16 |  |  |  |  |  | 3 (30) |  |  |
| 4q13.3 |  |  |  |  |  |  | 4 (31) |  |
| 4q27 |  |  |  |  |  |  | 4 (31) |  |
| 5p12-p15 | 1 (17) |  |  |  |  |  |  |  |
| 5q |  |  | 1 (14) |  |  |  |  |  |
| 6pter-q22 | 1 (17) |  |  |  |  |  |  |  |
| 6p21 | 3 (50) |  |  |  |  |  |  |  |
| 6p21.32 |  |  |  |  |  |  | 4 (31) |  |
| 6p25-p11.1 |  |  |  |  |  | 4 (40) |  |  |
| 6q11.1-q14 |  |  |  |  |  | 4 (40) |  |  |
| 6q14.1 |  |  |  |  |  |  | 4 (31) |  |
| 6q22-q24 |  |  | 1 (14) |  |  |  |  |  |
| 6q27 |  |  |  |  |  | 3 (30) |  |  |
| 7p12-p11.2 |  |  |  |  |  | 5 (50) |  |  |
| 7q | 1 (17) |  |  |  |  |  |  |  |
| 7q11-q31 | 1 (17) |  |  |  |  |  |  |  |
| 7q11.11-q31.1 |  |  |  |  | 2 (20) |  |  |  |
| 7q21.13 |  |  |  |  |  |  |  |  |
| 7q21.13-q21.2 |  |  |  |  |  |  |  |  |
| 7q21.2-q21.3 |  |  |  | 4 (24) |  |  |  |  |
| 7q22.1 |  |  |  |  |  |  |  |  |
| 7q22.2 |  |  |  |  |  |  |  |  |
| 7q22.2-q22.3 |  |  |  |  |  |  |  |  |
| 7q31.1-q31.2 |  |  |  | 4 (24) |  |  |  |  |
| 7q32.1-q34 |  |  |  |  | 2 (20) |  |  |  |
| 7q34 |  |  |  |  |  |  | 5 (38) |  |
| 7q35-qter |  |  |  |  | 2 (20) |  |  |  |
| 7q35-q36 |  |  |  |  |  | 6 (60) |  |  |
| 7q36.1 |  |  |  |  |  |  |  |  |
| 7q36.1-q36.3 |  |  |  |  |  |  |  |  |
| 8p23.3 |  |  |  |  |  | 4 (40) |  |  |
| 8q24.3 |  |  |  |  |  |  | 5 (38) |  |
| 9q | 1 (17) |  |  |  |  |  |  |  |
| 9q34 |  |  |  |  |  | 4 (40) |  |  |
| 10p15-p14 |  |  |  |  |  | 3 (30) |  |  |
| 10q |  |  | 1 (14) |  |  |  |  |  |
| 10q22-q23 |  |  | 1 (14) |  |  |  |  |  |
| 10q23-q25 |  |  | 1 (14) |  |  |  |  |  |
| 11p12-p14 |  |  | 1 (14) |  |  |  |  |  |
| 11p15 |  |  |  |  |  | 4 (40) |  |  |
| 11q12-q13 | 2 (33) |  |  |  |  |  |  |  |
| 11q14-q21 | 1 (17) |  |  |  |  |  |  |  |
| 11q14-q22 |  |  | 1 (14) |  |  |  |  |  |
| 11q23 | 1 (17) |  |  |  |  |  |  |  |
| 11q23-q24 | 1 (17) |  |  |  |  |  |  |  |
| 11q24.3 |  |  |  |  |  |  |  |  |
| 12q23-q24 | 2 (33) |  |  |  |  |  |  |  |
| 13q14.2 |  |  |  |  |  |  |  |  |
| 13q21-q33 |  |  | 1 (14) |  |  |  |  |  |
| 13q31-q32 |  |  | 1 (14) |  |  |  |  |  |
| 13q31-q34 |  |  | 2 (29) |  |  |  |  |  |
| 14q21-q23 | 1 (17) |  |  |  |  |  |  |  |
| 14q24-q32 | 1 (17) |  |  |  |  |  |  |  |
| 15q23-q24 | 1 (17) |  |  |  |  |  |  |  |
| 15q23-qter |  |  | 1 (14) |  |  |  |  |  |
| 16 | 1 (17) |  |  |  |  |  |  |  |
| 16p12-p13 | 1 (17) |  |  |  |  |  |  |  |
| 16p12.3 |  |  |  |  |  |  | 4 (31) |  |
| 16p13.3 |  |  |  |  |  | 4 (40) |  |  |
| 17 | 1 (17) |  |  |  |  |  |  |  |
| 17p12-q22 | 1 (17) |  |  |  |  |  |  |  |
| 17q12 |  |  |  |  |  | 6 (60) |  |  |
| 17q21-q24 |  |  | 2 (29) |  |  |  |  |  |
| 17q21-q25 | 1 (17) |  |  |  |  |  |  |  |
| 17q21.2-q21.31 |  |  |  | 3 (17) |  |  |  |  |
| 17q21.31-q21.33 |  |  |  |  | 2 (20) |  |  |  |
| 17q21.32-q21.33 |  |  |  |  |  |  |  |  |
| 17q21.33 |  |  |  |  |  |  |  |  |
| 17q23.1 |  |  |  |  |  |  |  |  |
| 17q23.2 |  |  |  |  |  |  |  |  |
| 17q23.2-q24.1 |  |  |  |  | 2 (20) |  |  |  |
| 17q24.2 |  |  |  |  |  |  |  |  |
| 17q25.1 |  |  |  |  |  |  |  |  |
| 17q25.3 |  |  |  |  |  |  |  |  |
| 18q22 |  |  | 1 (14) |  |  |  |  |  |
| 19 | 2 (33) |  |  |  |  |  |  |  |
| 19p13 | 1 (17) |  |  |  |  |  |  |  |
| 20 | 2 (33) |  |  |  |  |  |  |  |
| 20pter-qter |  |  |  |  | 5 (50) |  |  |  |
| 20q | 1 (17) |  |  |  |  |  |  |  |
| 20q11-q12 | 1 (17) |  |  |  |  |  |  |  |
| 21q21-qter |  |  | 1 (14) |  |  |  |  |  |
| 22 | 2 (33) |  |  |  |  |  |  |  |
| 22q11.21 |  |  |  |  |  | 4 (40) |  |  |
| 22q12-q13 |  |  | 1 (14) |  |  |  |  |  |
| X |  |  | 1 (14) |  |  |  |  |  |
| Xp21.3-p22.32 |  |  |  | 5 (29) |  |  |  |  |
| Xq28 |  |  |  | 5 (29) |  |  |  |  |
|  |  |  |  |  |  |  |  |  |
| *Recurring losses* |  |  |  |  |  |  |  |  |
| 1p |  |  | 2 (100) |  |  |  |  |  |
| 1p31-pter |  |  | 2 (29) |  |  |  |  |  |
| 1p32.3-p34.1 |  |  |  |  | 3 (30) |  |  |  |
| 1p34-pter |  |  | 2 (29) |  |  |  |  |  |
| 1p36.11-p36.32 |  |  |  |  | 3 (30) |  |  |  |
| 1p36.32 |  |  |  | 10 (58) |  |  |  |  |
| 1p21.3-p31.2 |  |  |  |  | 3 (30) |  |  |  |
| 1q21.1 |  |  |  |  |  |  | 7 (54) |  |
| 1q24.2-q24.3 |  |  |  | 3 (17) |  |  |  |  |
| 1q42.2-q42.3 |  |  |  | 3 (17) |  |  |  |  |
| 2p16.3 |  |  |  | 4 (24) |  |  |  |  |
| 2q14.3-q21.1 |  |  |  | 3 (17) |  |  |  |  |
| 3p24.2-p24.3 |  |  |  | 3 (17) |  |  |  |  |
| 3q21.1 |  |  |  |  |  |  | 6 (46) |  |
| 3q26.1 |  |  |  |  |  |  |  |  |
| 3q28-q29 |  |  |  | 3 (17) |  |  |  |  |
| 4q12 |  |  |  | 6 (35) |  |  |  |  |
| 4q21-q27 |  |  | 1 (14) |  |  |  |  |  |
| 4q31.3-q32.1 |  |  |  | 4 (24) |  |  |  |  |
| 5p13.2 |  |  |  |  |  |  | 8 (62) |  |
| 5p14.1-p14.2 |  |  |  | 4 (24) |  |  |  |  |
| 5q34-q35.3 |  |  |  | 6 (35) |  |  |  |  |
| 6q |  |  | 1 (25) |  |  |  |  |  |
| 6q13-q14 |  | 4 (57)*** |  |  |  |  |  |  |
| 6q16-q25 | 1 (17) |  |  |  |  | 4 (40) |  |  |
| 6q21 |  |  |  |  | 7 (70) |  |  |  |
| 6q21-qter |  |  | 1 (14) |  |  |  |  |  |
| 6q21-q22.1 |  |  |  | 6 (35) |  |  |  |  |
| 6q21-q23 |  | 4 (31)*** |  |  |  |  |  |  |
| 6q22.31 |  |  |  |  |  |  |  |  |
| 6q22.33-q23.2 |  |  |  | 8 (47) |  |  |  |  |
| 6q23.2 |  |  |  |  |  |  |  |  |
| 6q23.2-23.3 |  |  |  |  |  |  |  |  |
| 6q23.3-q24.3 |  |  |  |  |  |  |  |  |
| 6q24.3 |  | 7 (50)*** |  |  |  |  |  |  |
| 6q25-q27 | 1 (17) |  |  |  |  |  |  |  |
| 6q25.3 |  | 6 (67)*** |  | 5 (29) |  |  |  |  |
| 6q25.3-q26 |  |  |  |  |  |  |  |  |
| 6q26-q27 |  |  |  | 6 (35) |  |  |  |  |
| 6q27 |  | 5 (46)*** |  |  |  |  |  |  |
| 7p15.3-p22.3 |  |  |  |  | 3 (30) |  |  |  |
| 7p14.1 |  |  |  |  | 2 (20) |  |  |  |
| 7p15-p22 | 1 (17) |  |  |  |  |  |  |  |
| 7q22.1 |  |  |  | 5 (29) |  |  |  |  |
| 7q34 |  |  |  |  |  |  | 5 (38) |  |
| 8p11.23 |  |  |  |  |  |  | 6 (46) |  |
| 8p12-p21 |  |  | 1 (14) |  |  |  |  |  |
| 8p22 |  |  |  | 3 (17) |  |  |  |  |
| 8p23-p22 |  |  |  |  |  | 3 (30) |  |  |
| 8q21.2 |  |  |  | 3 (17) |  |  |  |  |
| 8q24.3 |  |  |  | 3 (17) |  |  |  |  |
| 9p21.3 |  |  |  |  |  |  |  |  |
| 9p21.3-p22.1 |  |  |  |  | 3 (30) |  |  |  |
| 9p11.2-p13.1 |  |  |  |  | 1 (10) |  |  |  |
| 10p12.1-p14 |  |  |  |  | 2 (20) |  |  |  |
| 11q13.5-q14 |  | 1 (10)*** |  |  |  |  |  |  |
| 11q21-q24 |  | 4 (31)*** |  |  |  |  |  |  |
| 11q23-q25 | 1 (17) |  |  |  |  |  |  |  |
| 11q23.1 |  |  |  | 5 (29) |  |  |  |  |
| 11q24.2-q25 |  |  |  |  | 2 (20) |  |  |  |
| 11q24-q25 |  |  |  |  |  | 4 (40) |  |  |
| 12q23-q24 |  |  | 1 (14) |  |  |  |  |  |
| 12q24 |  |  | 2 (29) |  |  |  |  |  |
| 12q24.13-q24.21 |  |  |  | 3 (17) |  |  |  |  |
| 12q24.31-q24.33 |  |  |  |  | 1 (10) |  |  |  |
| 13q12-q14 |  | 9 (60)*** |  |  |  |  |  |  |
| 13q14 |  | 8 (53)*** |  |  |  |  |  |  |
| 13q14.11 |  |  |  |  | 3 (30) |  |  |  |
| 13q14-q21 | 1 (17) |  |  |  |  |  |  |  |
| 13q14-q31 |  |  | 1 (14) |  |  |  |  |  |
| 13q14-q34 | 1 (17) |  |  |  |  |  |  |  |
| 13q21-q22 | 1 (17) | 1 (14)*** |  |  |  |  |  |  |
| 13q21-q31 |  |  | 1 (14) |  |  |  |  |  |
| 13q21.1 |  |  |  |  |  |  | 6 (46) |  |
| 13q31-q34 |  | 8 (53)*** |  |  |  |  |  |  |
| 14q11.1-11.2 |  |  |  |  |  |  | 4 (31) |  |
| 14q21.1 |  |  |  |  |  |  | 8 (62) |  |
| 14q24 |  |  | 1 (14) |  |  |  |  |  |
| 15q11.2 |  |  |  |  |  |  | 5 (38) |  |
| 15q11.2-q14 |  |  |  | 5 (29) |  |  |  |  |
| 15q24.2 |  |  |  |  |  |  | 9 (69) |  |
| 15q25.3 |  |  |  | 3 (17) | 2 (20) |  |  |  |
| 16p13.13-p13.2 |  |  |  |  | 3 (30) |  |  |  |
| 17p |  |  | 2 (29) |  |  |  |  |  |
| 17p11.2-p13.1 |  |  |  |  | 3 (30) |  |  |  |
| 17p12 |  | 2 (40)*** |  |  |  |  |  |  |
| 17p12-p13 |  |  | 1 (14) |  |  |  |  |  |
| 17p13 |  | 4 (36)*** | 1 (14) |  |  |  |  |  |
| 17p13.3 |  |  |  |  |  | 4 (40) |  |  |
| 18p11.31-pter |  |  |  |  | 3 (30) |  |  |  |
| 18q11.1-q12.1 |  |  |  |  | 2 (20) |  |  |  |
| 18q12.3-qter |  |  |  |  | 2 (20) |  |  |  |
| 19q13.32 |  |  |  |  |  |  | 9 (69) |  |
| 19q13.33 |  |  |  |  |  |  | 4 (31) |  |
| 20p13 |  |  |  |  |  |  | 5 (38) |  |
| 22q11.23 |  |  |  |  |  |  | 4 (31) |  |
| X | 2 (33) |  |  |  |  |  |  |  |

percentage in brackets, ***as reported in paper

Table S1C Recurrent copy number aberrations identified in ENKTL using Oncoscan-molecular inversion probe assay.[[8](#_ENREF_8)]

| **Recurrent copy number gains in ENKTL (n=29)** | | | | |
| --- | --- | --- | --- | --- |
| chromosome | start position | end position | band | No. of cases (n=29) |
| chr1 | 89474818 | 89823863 | chr1p22.2 | 4 |
| chr1 | 89823864 | 90049614 | chr1p22.2 | 5 |
| chr1 | 90049615 | 90221440 | chr1p22.2 | 4 |
| chr1 | 145382341 | 145676265 | chr1q21.1 | 4 |
| chr1 | 146822802 | 146969410 | chr1q21.1 | 4 |
| chr1 | 147091689 | 150199410 | chr1q21.2 | 4 |
| chr1 | 152624922 | 152888177 | chr1q21.3 | 4 |
| chr1 | 154317153 | 154635447 | chr1q21.3 | 4 |
| chr1 | 154635448 | 154900720 | chr1q21.3 | 4 |
| chr1 | 154900721 | 160152056 | chr1q21.3-q23.2 | 4 |
| chr1 | 160152057 | 160372108 | chr1q23.2 | 4 |
| chr1 | 160372109 | 160481661 | chr1q23.2 | 5 |
| chr1 | 160481662 | 160681774 | chr1q23.2-q23.3 | 6 |
| chr1 | 160681775 | 160723255 | chr1q23.3 | 8 |
| chr1 | 160723256 | 160902162 | chr1q23.3 | 6 |
| chr1 | 160902163 | 161388959 | chr1q23.3 | 5 |
| chr1 | 161388960 | 161508762 | chr1q23.3 | 6 |
| chr1 | 161508763 | 161622700 | chr1q23.3 | 6 |
| chr1 | 161622701 | 161764792 | chr1q23.3 | 5 |
| chr1 | 161764793 | 163288543 | chr1q23.3 | 4 |
| chr1 | 163288544 | 163492131 | chr1q23.3 | 4 |
| chr1 | 163492132 | 163734858 | chr1q23.3 | 5 |
| chr1 | 163734859 | 166664570 | chr1q23.3-q24.1 | 4 |
| chr1 | 167553649 | 172358166 | chr1q24.2-q24.3 | 4 |
| chr1 | 172358167 | 172522890 | chr1q24.3 | 5 |
| chr1 | 172522891 | 172538552 | chr1q24.3 | 5 |
| chr1 | 172538553 | 172613240 | chr1q24.3 | 6 |
| chr1 | 172613241 | 172664441 | chr1q24.3 | 5 |
| chr1 | 172664442 | 172898376 | chr1q24.3 | 5 |
| chr1 | 172898377 | 173019052 | chr1q24.3-q25.1 | 4 |
| chr1 | 173019053 | 173302305 | chr1q25.1 | 4 |
| chr1 | 173302306 | 173322659 | chr1q25.1 | 5 |
| chr1 | 173322660 | 173394053 | chr1q25.1 | 5 |
| chr1 | 173394054 | 173893017 | chr1q25.1 | 6 |
| chr1 | 173893018 | 174005027 | chr1q25.1 | 5 |
| chr1 | 174005028 | 174576479 | chr1q25.1 | 4 |
| chr1 | 174576480 | 174983632 | chr1q25.1 | 4 |
| chr1 | 174983633 | 175193107 | chr1q25.1 | 4 |
| chr1 | 175193108 | 175889626 | chr1q25.1 | 4 |
| chr1 | 175889627 | 176291702 | chr1q25.1-q25.2 | 4 |
| chr1 | 176291703 | 176422272 | chr1q25.2 | 4 |
| chr1 | 176422273 | 176684850 | chr1q25.2 | 4 |
| chr1 | 176684851 | 178127876 | chr1q25.2 | 4 |
| chr1 | 182182087 | 182443461 | chr1q25.3 | 4 |
| chr1 | 183413886 | 183667179 | chr1q25.3 | 4 |
| chr1 | 184677464 | 185001674 | chr1q25.3 | 4 |
| chr1 | 196922021 | 197050843 | chr1q31.3 | 4 |
| chr1 | 197050844 | 198013968 | chr1q31.3 | 4 |
| chr1 | 198013969 | 198172402 | chr1q31.3 | 5 |
| chr1 | 198172403 | 199139428 | chr1q31.3-q32.1 | 5 |
| chr1 | 199139429 | 200251049 | chr1q32.1 | 5 |
| chr1 | 200251050 | 200790619 | chr1q32.1 | 4 |
| chr1 | 201535727 | 203207519 | chr1q32.1 | 4 |
| chr1 | 203207520 | 203301964 | chr1q32.1 | 5 |
| chr1 | 203301965 | 204554317 | chr1q32.1 | 4 |
| chr1 | 204980360 | 208548173 | chr1q32.1-q32.2 | 4 |
| chr1 | 208548174 | 214574034 | chr1q32.2-q41 | 5 |
| chr1 | 214574035 | 214814777 | chr1q41 | 5 |
| chr1 | 214814778 | 221373328 | chr1q41 | 5 |
| chr1 | 221373329 | 221866184 | chr1q41 | 5 |
| chr1 | 221866185 | 222109517 | chr1q41 | 6 |
| chr1 | 222109518 | 222420675 | chr1q41 | 5 |
| chr1 | 222420676 | 223532569 | chr1q41 | 5 |
| chr1 | 223532570 | 224891567 | chr1q41-q42.12 | 6 |
| chr1 | 224891568 | 226544419 | chr1q42.12 | 5 |
| chr1 | 226544420 | 226849760 | chr1q42.12 | 4 |
| chr1 | 226849761 | 228057760 | chr1q42.12-q42.13 | 5 |
| chr1 | 228057761 | 228331039 | chr1q42.13 | 5 |
| chr1 | 228331040 | 228482284 | chr1q42.13 | 5 |
| chr1 | 228482285 | 228603161 | chr1q42.13 | 4 |
| chr1 | 228603162 | 229195148 | chr1q42.13 | 5 |
| chr1 | 229195149 | 229435891 | chr1q42.13 | 5 |
| chr1 | 229435892 | 229810259 | chr1q42.13 | 5 |
| chr1 | 229810260 | 230070287 | chr1q42.13 | 6 |
| chr1 | 230070288 | 231060735 | chr1q42.13-q42.2 | 5 |
| chr1 | 231060736 | 231313858 | chr1q42.2 | 6 |
| chr1 | 231313859 | 235253626 | chr1q42.2-q42.3 | 5 |
| chr1 | 235253627 | 235499262 | chr1q42.3 | 6 |
| chr1 | 235499263 | 237346856 | chr1q42.3-q43 | 5 |
| chr1 | 237346857 | 237840874 | chr1q43 | 5 |
| chr1 | 237840875 | 244048655 | chr1q43-q44 | 5 |
| chr1 | 244048656 | 244316322 | chr1q44 | 6 |
| chr1 | 244316323 | 248845096 | chr1q44 | 5 |
| chr1 | 248845097 | 249212877 | chr1q44 | 6 |
| chr2 | 8204300 | 8260500 | chr2p25.1 | 4 |
| chr2 | 8260501 | 8649156 | chr2p25.1 | 5 |
| chr2 | 8649157 | 8751462 | chr2p25.1 | 4 |
| chr2 | 99055922 | 99082358 | chr2q11.2 | 4 |
| chr2 | 135311142 | 135597627 | chr2q21.3 | 4 |
| chr2 | 136659598 | 136721602 | chr2q21.3 | 4 |
| chr2 | 136721603 | 136873152 | chr2q21.3-q22.1 | 5 |
| chr2 | 136873153 | 137091556 | chr2q22.1 | 6 |
| chr2 | 137091557 | 137142499 | chr2q22.1 | 5 |
| chr2 | 181988909 | 182428634 | chr2q31.3 | 4 |
| chr2 | 191479670 | 191716705 | chr2q32.2 | 4 |
| chr2 | 191729154 | 191770077 | chr2q32.2 | 4 |
| chr2 | 191770078 | 191802682 | chr2q32.2 | 5 |
| chr2 | 191802683 | 191996517 | chr2q32.2-q32.3 | 6 |
| chr2 | 191996518 | 192037581 | chr2q32.3 | 5 |
| chr2 | 204351363 | 204569716 | chr2q33.2 | 4 |
| chr2 | 204569717 | 204798019 | chr2q33.2 | 4 |
| chr2 | 213438055 | 214177390 | chr2q34 | 4 |
| chr3 | 46108247 | 46477221 | chr3p21.31 | 4 |
| chr3 | 59273427 | 59340344 | chr3p14.2 | 5 |
| chr3 | 59340345 | 59625964 | chr3p14.2 | 6 |
| chr3 | 59625965 | 59655529 | chr3p14.2 | 4 |
| chr4 | 143268885 | 143334047 | chr4q31.21 | 4 |
| chr5 | 156622572 | 156664821 | chr5q33.3 | 4 |
| chr6 | 204909 | 352526 | chr6p25.3 | 4 |
| chr6 | 26145217 | 26158992 | chr6p22.2 | 4 |
| chr6 | 26158993 | 26187124 | chr6p22.2 | 5 |
| chr6 | 26187125 | 26374657 | chr6p22.2 | 6 |
| chr6 | 32340236 | 32561715 | chr6p21.32 | 5 |
| chr6 | 32561716 | 32572525 | chr6p21.32 | 8 |
| chr6 | 32572526 | 32582215 | chr6p21.32 | 9 |
| chr6 | 32582216 | 32586695 | chr6p21.32 | 8 |
| chr6 | 32586696 | 32600525 | chr6p21.32 | 7 |
| chr6 | 32600526 | 32658178 | chr6p21.32 | 4 |
| chr7 | 50253721 | 50269671 | chr7p12.2 | 4 |
| chr7 | 50269672 | 50425290 | chr7p12.2 | 5 |
| chr7 | 50425291 | 50452551 | chr7p12.2 | 4 |
| chr7 | 149642556 | 149883124 | chr7q36.1 | 4 |
| chr7 | 150103137 | 150153812 | chr7q36.1 | 4 |
| chr7 | 150153813 | 150414571 | chr7q36.1 | 5 |
| chr7 | 150414572 | 150483511 | chr7q36.1 | 4 |
| chr7 | 154763487 | 154953587 | chr7q36.2 | 4 |
| chr7 | 158401375 | 158644668 | chr7q36.3 | 4 |
| chr7 | 158644669 | 158670242 | chr7q36.3 | 4 |
| chr7 | 158670243 | 158739969 | chr7q36.3 | 4 |
| chr8 | 66677039 | 66917136 | chr8q13.1 | 4 |
| chr8 | 125637207 | 125799894 | chr8q24.13 | 4 |
| chr10 | 3818058 | 3946950 | chr10p15.1 | 4 |
| chr10 | 6898219 | 7122365 | chr10p14 | 4 |
| chr10 | 126425866 | 126512247 | chr10q26.13 | 4 |
| chr11 | 128336438 | 128582943 | chr11q24.3 | 4 |
| chr12 | 9750669 | 9788571 | chr12p13.31 | 4 |
| chr12 | 9788572 | 9943699 | chr12p13.31 | 5 |
| chr12 | 9943700 | 10122045 | chr12p13.31-p13.2 | 4 |
| chr14 | 106475814 | 106531399 | chr14q32.33 | 5 |
| chr14 | 106531400 | 106545057 | chr14q32.33 | 5 |
| chr14 | 106545058 | 106572891 | chr14q32.33 | 4 |
| chr16 | 50767647 | 50854458 | chr16q12.1 | 7 |
| chr17 | 44810652 | 45229721 | chr17q21.31-q21.32 | 4 |
| chr17 | 45229722 | 46230837 | chr17q21.32 | 5 |
| chr17 | 46230838 | 46297150 | chr17q21.32 | 6 |
| chr17 | 46297151 | 46549890 | chr17q21.32 | 7 |
| chr17 | 46549891 | 46603061 | chr17q21.32 | 5 |
| chr17 | 46603062 | 46632250 | chr17q21.32 | 4 |
| chr17 | 46632251 | 47544909 | chr17q21.32-q21.33 | 4 |
| chr17 | 49193326 | 49283088 | chr17q21.33 | 4 |
| chr17 | 77951619 | 78735891 | chr17q25.3 | 4 |
| chr19 | 1246308 | 1265404 | chr19p13.3 | 4 |
| chr20 | 37349737 | 37598943 | chr20q11.23 | 4 |
| chr20 | 42192083 | 42321221 | chr20q13.12 | 4 |
| chr20 | 60591084 | 60778376 | chr20q13.33 | 4 |
| chr20 | 61269348 | 61652929 | chr20q13.33 | 4 |
|  |  |  |  |  |
| **Recurrent copy number loss in ENKTL (n=29)** | | | | |
| chromosome | start position | end position | band | No. of cases (n=29) |
| chr1 | 43623644 | 43683611 | chr1p34.2 | 4 |
| chr1 | 43683612 | 43731340 | chr1p34.2 | 5 |
| chr1 | 43731341 | 43784664 | chr1p34.2 | 4 |
| chr3 | 162500864 | 162619269 | chr3q26.1 | 4 |
| chr6 | 110665556 | 110911298 | chr6q21 | 4 |
| chr6 | 113476791 | 113506305 | chr6q21 | 4 |
| chr6 | 113506306 | 113608615 | chr6q21 | 5 |
| chr6 | 113608616 | 113732394 | chr6q21 | 6 |
| chr6 | 113732395 | 113876687 | chr6q21 | 7 |
| chr6 | 113876688 | 113973395 | chr6q21 | 6 |
| chr6 | 113973396 | 114004813 | chr6q21 | 4 |
| chr6 | 115804926 | 116787016 | chr6q22.1 | 4 |
| chr6 | 116787017 | 117075631 | chr6q22.1 | 5 |
| chr6 | 117075632 | 119886327 | chr6q22.1-q22.31 | 4 |
| chr6 | 119886328 | 120377150 | chr6q22.31 | 5 |
| chr6 | 120377151 | 125372425 | chr6q22.31 | 4 |
| chr6 | 125854253 | 126090607 | chr6q22.31 | 4 |
| chr6 | 129293043 | 129556516 | chr6q22.33 | 4 |
| chr6 | 132429242 | 132712306 | chr6q23.2 | 4 |
| chr6 | 140545114 | 140783204 | chr6q24.1 | 4 |
| chr6 | 141809473 | 142048983 | chr6q24.1 | 4 |
| chr6 | 143488404 | 143762244 | chr6q24.2 | 4 |
| chr6 | 147836218 | 148544121 | chr6q24.3 | 4 |
| chr6 | 148981394 | 149273771 | chr6q24.3-q25.1 | 4 |
| chr7 | 27178790 | 27264177 | chr7p15.2 | 5 |
| chr7 | 27264178 | 27267904 | chr7p15.2 | 4 |
| chr7 | 110629643 | 110743601 | chr7q31.1 | 4 |
| chr8 | 11064269 | 11622292 | chr8p23.1 | 4 |
| chr8 | 11739398 | 15356233 | chr8p23.1-p22 | 4 |
| chr8 | 15356234 | 15476921 | chr8p22 | 5 |
| chr8 | 15476922 | 15842538 | chr8p22 | 4 |
| chr8 | 16796082 | 17002724 | chr8p22 | 4 |
| chr8 | 17344517 | 17406182 | chr8p22 | 4 |
| chr8 | 17406183 | 17545705 | chr8p22 | 5 |
| chr8 | 17545706 | 17582136 | chr8p22 | 6 |
| chr8 | 17582137 | 17632942 | chr8p22 | 7 |
| chr8 | 17632943 | 17787535 | chr8p22 | 6 |
| chr8 | 17787536 | 18030249 | chr8p22 | 5 |
| chr8 | 18030250 | 18565804 | chr8p22 | 4 |
| chr8 | 18565805 | 18624827 | chr8p22 | 5 |
| chr8 | 18624828 | 18645146 | chr8p22 | 6 |
| chr8 | 18645147 | 18766525 | chr8p22 | 5 |
| chr8 | 18766526 | 19303953 | chr8p22-p21.3 | 4 |
| chr8 | 19303954 | 19607875 | chr8p21.3 | 4 |
| chr8 | 19607876 | 21662444 | chr8p21.3 | 4 |
| chr9 | 4185934 | 4331716 | chr9p24.2 | 4 |
| chr10 | 602716 | 636021 | chr10p15.3 | 4 |
| chr10 | 12608676 | 12797006 | chr10p13 | 4 |
| chr10 | 21502882 | 21625571 | chr10p12.31 | 4 |
| chr10 | 69551465 | 69664958 | chr10q21.3 | 4 |
| chr11 | 93951906 | 94319284 | chr11q21 | 4 |
| chr11 | 107219307 | 107396157 | chr11q22.3 | 4 |
| chr11 | 107396158 | 107651608 | chr11q22.3 | 5 |
| chr11 | 112268530 | 112570796 | chr11q23.1-q23.2 | 4 |
| chr11 | 113991812 | 114051191 | chr11q23.2 | 4 |
| chr11 | 114051192 | 114074755 | chr11q23.2 | 4 |
| chr12 | 52414095 | 52437490 | chr12q13.13 | 4 |
| chr13 | 100862964 | 101095826 | chr13q32.3 | 4 |
| chr13 | 101755480 | 101910020 | chr13q33.1 | 4 |
| chr14 | 22322690 | 22333938 | chr14q11.2 | 5 |
| chr14 | 22333939 | 22417381 | chr14q11.2 | 6 |
| chr14 | 22417382 | 22428629 | chr14q11.2 | 8 |
| chr14 | 22428630 | 22461776 | chr14q11.2 | 9 |
| chr14 | 22461777 | 22471905 | chr14q11.2 | 9 |
| chr14 | 22471906 | 22516202 | chr14q11.2 | 10 |
| chr14 | 22516203 | 22542031 | chr14q11.2 | 10 |
| chr14 | 22542032 | 22553659 | chr14q11.2 | 10 |
| chr14 | 22553660 | 22573442 | chr14q11.2 | 10 |
| chr14 | 22573443 | 22617503 | chr14q11.2 | 10 |
| chr14 | 22617504 | 22701815 | chr14q11.2 | 10 |
| chr14 | 22701816 | 22763062 | chr14q11.2 | 11 |
| chr14 | 22763063 | 22890410 | chr14q11.2 | 12 |
| chr14 | 22890411 | 22911701 | chr14q11.2 | 12 |
| chr14 | 22911702 | 22913993 | chr14q11.2 | 11 |
| chr14 | 22913994 | 22930807 | chr14q11.2 | 11 |
| chr14 | 22930808 | 22942148 | chr14q11.2 | 10 |
| chr14 | 22942149 | 22950247 | chr14q11.2 | 9 |
| chr14 | 22950248 | 22961853 | chr14q11.2 | 8 |
| chr14 | 22961854 | 22988570 | chr14q11.2 | 7 |
| chr14 | 22988571 | 23000061 | chr14q11.2 | 4 |
| chr16 | 916723 | 1297144 | chr16p13.3 | 4 |
| chr16 | 32564454 | 33836545 | chr16p11.2 | 5 |
| chr17 | 7605781 | 7620248 | chr17p13.1 | 4 |
| chr17 | 8932082 | 9181458 | chr17p13.1 | 4 |
| chr17 | 9895167 | 10020803 | chr17p13.1 | 4 |
| chr17 | 10020804 | 10110571 | chr17p13.1 | 4 |
| chr17 | 10110572 | 10188066 | chr17p13.1 | 4 |
| chr17 | 10188067 | 12562436 | chr17p13.1-p12 | 4 |
| chr18 | 14935915 | 15194970 | chr18p11.21 | 4 |
| chr18 | 55786931 | 56037487 | chr18q21.31 | 4 |
| chr22 | 24346428 | 24347757 | chr22q11.23 | 10 |
| chr22 | 24347758 | 24382737 | chr22q11.23 | 12 |
| chr22 | 24382738 | 24388237 | chr22q11.23 | 12 |
| chr22 | 24388238 | 24394087 | chr22q11.23 | 8 |
| chr22 | 24394088 | 24398767 | chr22q11.23 | 4 |

Table S3 List of genes which are upregulated in ENKTL tumor compared to normal controls (p-value less than 0.05 and fold change greater than 1.5)

| symbol | p-value | False discovery rate | Fold change |
| --- | --- | --- | --- |
| KLRC2 | 1.01E-07 | 0.000284272 | 22.05099688 |
| HIST2H2AA3 | 3.25E-05 | 0.006509799 | 7.335028693 |
| KLRC1 | 3.08E-08 | 0.000171864 | 6.49151986 |
| FCGR3A | 3.68E-08 | 0.000171864 | 6.310203863 |
| CCL4 | 6.31E-08 | 0.000221217 | 6.181383076 |
| GZMA | 1.99E-08 | 0.000171864 | 6.043814329 |
| GZMK | 6.62E-05 | 0.010785469 | 5.772819335 |
| KLRC3 | 6.22E-07 | 0.000856998 | 5.592590257 |
| CXCL9 | 2.64E-06 | 0.002179586 | 5.470501657 |
| KLRC4-KLRK1 | 1.39E-05 | 0.004065224 | 5.417159939 |
| LGALS9C | 0.000453399 | 0.029841802 | 5.194712444 |
| CENPS | 0.003697341 | 0.077431468 | 5.049641597 |
| CXCL10 | 1.84E-05 | 0.004604994 | 4.628433513 |
| GBP5 | 1.80E-05 | 0.004604994 | 4.628170148 |
| KLRC4 | 0.011092618 | 0.121492152 | 4.485660646 |
| IFNG | 3.23E-05 | 0.006509799 | 4.156327915 |
| APOBEC3G | 0.000379421 | 0.026595986 | 3.886993007 |
| SENP3 | 0.028523762 | 0.172737183 | 3.695453769 |
| SNORD102 | 0.023451075 | 0.160451934 | 3.680957493 |
| FCER1G | 2.76E-05 | 0.006047866 | 3.601789015 |
| S100A8 | 2.40E-05 | 0.005524969 | 3.591691548 |
| SPECC1L | 0.020007792 | 0.152111624 | 3.492780008 |
| RTEL1 | 2.49E-05 | 0.005627349 | 3.394576633 |
| MIR29A | 4.11E-07 | 0.000660361 | 3.372683871 |
| HIST1H2BI | 0.002344335 | 0.06385773 | 3.317449642 |
| HSPE1 | 0.002639173 | 0.06727121 | 3.291819139 |
| SNORD12C | 0.00129797 | 0.048101984 | 3.275641976 |
| MIR18A | 0.006807305 | 0.102067568 | 3.247560521 |
| HIST1H3G | 0.000634499 | 0.03529835 | 3.191443885 |
| CLEC2B | 0.001750589 | 0.054233003 | 3.174916231 |
| GYG1 | 1.31E-05 | 0.003915713 | 3.165288505 |
| RPSA | 0.031163314 | 0.177885038 | 3.112404611 |
| HSPA6 | 3.55E-06 | 0.002290477 | 3.104558087 |
| SNORD61 | 0.025834818 | 0.167059153 | 3.011499278 |
| CBWD5 | 0.003834509 | 0.078706967 | 2.981410645 |
| CCL3 | 0.000487067 | 0.030591988 | 2.961158263 |
| CXCL11 | 4.21E-05 | 0.007768903 | 2.953540254 |
| ZNF594 | 0.000887965 | 0.041576067 | 2.931102635 |
| EEF1E1 | 0.000160348 | 0.017292007 | 2.925695923 |
| COX7B | 0.002093388 | 0.060386187 | 2.872691194 |
| SNORD45C | 9.06E-06 | 0.003344015 | 2.839719502 |
| OR2T8 | 5.55E-06 | 0.002432951 | 2.793922714 |
| ANKRD22 | 0.000369487 | 0.026294033 | 2.761991316 |
| ASPM | 1.97E-05 | 0.004750848 | 2.759399459 |
| CD163 | 1.92E-06 | 0.001795747 | 2.748437798 |
| MSR1 | 2.56E-06 | 0.002179586 | 2.743900323 |
| GZMB | 3.25E-06 | 0.00227836 | 2.725631935 |
| CKLF | 0.004679635 | 0.086095644 | 2.724628865 |
| CENPF | 1.60E-05 | 0.00442789 | 2.722153319 |
| DDIT4 | 7.19E-05 | 0.011323848 | 2.712817133 |
| SNRPD1 | 0.000432927 | 0.029060616 | 2.695966259 |
| HIST2H4A | 0.025078546 | 0.165061925 | 2.691948217 |
| GPR171 | 4.15E-06 | 0.002315341 | 2.690297155 |
| SPDYE6 | 0.039790641 | 0.19621037 | 2.627178319 |
| GNLY | 1.09E-05 | 0.003483731 | 2.611518835 |
| RRN3P1 | 0.009379476 | 0.11359998 | 2.595984305 |
| S100A9 | 0.000267795 | 0.022891935 | 2.570343414 |
| ATP8B4 | 1.05E-06 | 0.001221048 | 2.56956518 |
| F2R | 3.34E-05 | 0.006589845 | 2.568556604 |
| NME1 | 0.006081259 | 0.097010149 | 2.561099804 |
| DTHD1 | 0.001591253 | 0.051919936 | 2.542169598 |
| ELK2AP | 0.023530466 | 0.160838129 | 2.537067994 |
| HIST1H2AC | 0.01331101 | 0.129769705 | 2.53544438 |
| ARHGAP11A | 1.04E-05 | 0.003458515 | 2.532726046 |
| APOBEC3A | 0.002489593 | 0.065605593 | 2.518139819 |
| HIST2H2BA | 0.000201534 | 0.019789102 | 2.510488847 |
| SKA2 | 0.001619515 | 0.05263449 | 2.504252549 |
| RPL23AP82 | 0.001253132 | 0.047480916 | 2.479410045 |
| MS4A4A | 0.000234874 | 0.021223109 | 2.471321131 |
| APOBEC3H | 3.76E-06 | 0.002290477 | 2.465362884 |
| PSTPIP2 | 2.94E-05 | 0.006234823 | 2.457344072 |
| SNX10 | 1.66E-06 | 0.001665223 | 2.44575168 |
| SIGLEC17P | 4.69E-06 | 0.002432951 | 2.432993324 |
| PILRB | 0.012818015 | 0.128050442 | 2.431926804 |
| PLEK | 0.000485522 | 0.030591988 | 2.428488089 |
| PRF1 | 1.18E-06 | 0.001275045 | 2.427679508 |
| TOP2A | 0.000273921 | 0.023142424 | 2.424579617 |
| TLR8 | 0.000115363 | 0.0157134 | 2.411868238 |
| RGS1 | 0.004975663 | 0.08817109 | 2.411442659 |
| GBP2 | 0.000288995 | 0.024260368 | 2.410911836 |
| TPX2 | 3.74E-05 | 0.007178299 | 2.399782549 |
| NCAPG | 0.000123293 | 0.0157134 | 2.385002307 |
| ZCRB1 | 0.004450327 | 0.083745167 | 2.371945625 |
| CENPW | 1.15E-05 | 0.003590256 | 2.367933257 |
| GIMAP2 | 0.01638664 | 0.141284174 | 2.366986159 |
| SLFN12L | 0.000360667 | 0.026063263 | 2.363676286 |
| KLRD1 | 2.55E-05 | 0.005680693 | 2.35818673 |
| ZNHIT2 | 0.000315587 | 0.024995985 | 2.355891082 |
| XCL1 | 4.17E-05 | 0.007768903 | 2.343048492 |
| GBP1 | 0.006263775 | 0.097861952 | 2.341704645 |
| SAMSN1 | 0.000368514 | 0.026294033 | 2.340249233 |
| CTSL | 0.000113691 | 0.0157134 | 2.334374478 |
| C8orf76 | 8.45E-05 | 0.013011696 | 2.330631072 |
| IDO1 | 9.94E-06 | 0.003398762 | 2.32750664 |
| HCRP1 | 0.004885175 | 0.087398462 | 2.319198456 |
| SH2D1A | 0.00267187 | 0.06773518 | 2.308471026 |
| FCGR2C | 0.021531511 | 0.155429007 | 2.298457009 |
| HCP5 | 0.009734928 | 0.115169784 | 2.294005525 |
| KPNA2 | 0.000483493 | 0.030591988 | 2.277345445 |
| SLC39A8 | 6.72E-07 | 0.000856998 | 2.2654335 |
| KIR2DL1 | 0.004432088 | 0.083626457 | 2.251705759 |
| CD96 | 0.004805038 | 0.086807901 | 2.247053972 |
| LMNB1 | 5.75E-05 | 0.009516077 | 2.244657351 |
| FCGR2A | 0.002335946 | 0.063836563 | 2.238781557 |
| SAMD3 | 2.25E-05 | 0.005248124 | 2.227357858 |
| SNORD28 | 0.038508405 | 0.193988453 | 2.220040283 |
| POTEC | 0.000816836 | 0.039900371 | 2.218000679 |
| KIF11 | 9.73E-05 | 0.014347673 | 2.208331926 |
| NKG7 | 5.42E-06 | 0.002432951 | 2.206498147 |
| HPRT1 | 0.000247945 | 0.021590086 | 2.200739628 |
| UBE2T | 0.000292683 | 0.024279213 | 2.197732051 |
| CCNB1 | 4.29E-06 | 0.002315341 | 2.190464989 |
| MAD2L1 | 0.000201854 | 0.019789102 | 2.185947963 |
| FPR2 | 0.000128447 | 0.015795908 | 2.184219528 |
| SMC4 | 0.000754347 | 0.037634757 | 2.169438134 |
| BUB1 | 0.00011556 | 0.0157134 | 2.169034312 |
| KIR2DL4 | 0.000224261 | 0.020635117 | 2.165173541 |
| TARP | 0.004830597 | 0.086822121 | 2.158723477 |
| EOMES | 0.001805383 | 0.054665395 | 2.151932466 |
| TIMP1 | 0.00185536 | 0.056057569 | 2.150707743 |
| MRC1 | 0.000341388 | 0.025870261 | 2.150138358 |
| HSPD1 | 0.003015629 | 0.070815413 | 2.147706033 |
| CTSW | 7.28E-06 | 0.002836338 | 2.147264874 |
| LOC101060256 | 0.005513287 | 0.092334068 | 2.146512067 |
| CDKN3 | 0.000214725 | 0.020339762 | 2.143515428 |
| ANP32E | 0.000713658 | 0.0371044 | 2.138408195 |
| HSP90AB3P | 0.000149533 | 0.017052244 | 2.138113401 |
| KIF20B | 3.03E-05 | 0.006349125 | 2.132852468 |
| HCST | 0.001332031 | 0.048378372 | 2.132006877 |
| CKAP2 | 0.000535861 | 0.032104068 | 2.128474707 |
| RAD51AP1 | 0.000116084 | 0.0157134 | 2.121004847 |
| ANKRD36BP1 | 0.027470447 | 0.170591994 | 2.109074726 |
| CCL5 | 0.009828547 | 0.115788791 | 2.105036961 |
| HIST1H2BK | 0.00712106 | 0.103636398 | 2.093607041 |
| ANXA1 | 0.010419817 | 0.117791026 | 2.090649718 |
| HIST1H1C | 0.004176126 | 0.081997295 | 2.086236425 |
| SNORD79 | 0.034427631 | 0.184357857 | 2.082619073 |
| SNORD46 | 0.042548519 | 0.199478309 | 2.08236742 |
| SPINK7 | 0.002147924 | 0.06157921 | 2.077964954 |
| HIST1H3D | 0.033276602 | 0.181734444 | 2.074376914 |
| NCAM1 | 0.000125632 | 0.015781052 | 2.071029429 |
| C1QA | 0.00058205 | 0.033579811 | 2.069746391 |
| SRGN | 0.002414019 | 0.064832444 | 2.067751437 |
| TYMS | 9.82E-05 | 0.014347673 | 2.06514047 |
| SERPINB12 | 0.021533184 | 0.155429007 | 2.062106154 |
| VEGFD | 0.000876035 | 0.041212536 | 2.05674016 |
| DZIP3 | 0.001781907 | 0.054282678 | 2.053161722 |
| KIF15 | 0.000193326 | 0.019789102 | 2.052562404 |
| KCNRG | 0.030883416 | 0.177588886 | 2.04674848 |
| CXCL8 | 0.000596339 | 0.033800589 | 2.037215825 |
| PTPN22 | 0.007634083 | 0.105510947 | 2.032622586 |
| SLFN13 | 0.000243692 | 0.021352309 | 2.02959861 |
| GPRIN3 | 0.002721112 | 0.067999805 | 2.0246423 |
| ADORA3 | 0.000606745 | 0.034161045 | 2.020398274 |
| CLEC12A | 0.000142408 | 0.01649964 | 2.020064616 |
| IL2RB | 0.000199786 | 0.019789102 | 2.016559701 |
| DUT | 0.001157458 | 0.046365797 | 2.014810543 |
| DTL | 2.82E-05 | 0.006089883 | 2.010752554 |
| SEPT7 | 0.010169318 | 0.117271605 | 2.010184765 |
| FUT5 | 0.000558316 | 0.032749606 | 2.004625104 |
| WARS | 0.001384542 | 0.049275194 | 2.001646463 |
| GTPBP8 | 0.00102565 | 0.043376688 | 1.993191534 |
| DSCC1 | 4.24E-07 | 0.000660361 | 1.991773843 |
| SCD | 0.0004503 | 0.029777617 | 1.989849238 |
| SAMD9L | 0.012765475 | 0.127995742 | 1.985107016 |
| PSMA5 | 0.001656762 | 0.052906438 | 1.982703418 |
| CENPE | 9.66E-06 | 0.003384096 | 1.978324677 |
| MINOS1 | 9.49E-06 | 0.003384096 | 1.973656244 |
| LGALS1 | 0.000433238 | 0.029060616 | 1.972224297 |
| NEDD8 | 0.039037156 | 0.194867383 | 1.969921939 |
| TVP23B | 0.002624793 | 0.06727121 | 1.967721349 |
| PYHIN1 | 0.024089902 | 0.162679128 | 1.963781303 |
| CALU | 0.000150827 | 0.017052244 | 1.960611906 |
| C14orf2 | 0.002086497 | 0.060311512 | 1.955181078 |
| PRR11 | 0.000109989 | 0.015700506 | 1.954464795 |
| VSIG4 | 3.98E-06 | 0.002315341 | 1.952305955 |
| CD63 | 0.002266405 | 0.063167492 | 1.948762136 |
| SLC27A2 | 1.75E-05 | 0.004604994 | 1.946752758 |
| ARHGDIB | 0.028794531 | 0.172810596 | 1.943340455 |
| SMC3 | 0.010403531 | 0.117791026 | 1.942159726 |
| HIST1H2AB | 6.71E-06 | 0.002742791 | 1.940587306 |
| CLSPN | 1.47E-05 | 0.00421503 | 1.93962751 |
| HELLS | 0.000568926 | 0.033232963 | 1.934668526 |
| GNGT2 | 5.01E-06 | 0.002432951 | 1.930491008 |
| GCH1 | 0.000110873 | 0.015700506 | 1.929385484 |
| PARP8 | 0.008550245 | 0.109646798 | 1.923025842 |
| CCR1 | 0.000963719 | 0.042439677 | 1.921070906 |
| PTTG1 | 9.39E-05 | 0.01415511 | 1.917713482 |
| IFI30 | 0.000859492 | 0.040916856 | 1.916549935 |
| HSP90AA1 | 0.024939894 | 0.164661107 | 1.911832828 |
| RRM2 | 0.002952436 | 0.070512576 | 1.91138254 |
| PWP2 | 0.012403894 | 0.126467664 | 1.906421091 |
| SH2D1B | 0.000860994 | 0.040916856 | 1.904547036 |
| SLAMF7 | 0.005136628 | 0.089578549 | 1.903841693 |
| CLEC7A | 0.000699173 | 0.036849124 | 1.902945251 |
| HTATSF1 | 1.90E-05 | 0.004673089 | 1.898557633 |
| NUF2 | 0.001463588 | 0.049801877 | 1.89677868 |
| PA2G4 | 0.005388807 | 0.09222852 | 1.896097621 |
| PAIP2 | 0.019825675 | 0.151761645 | 1.895493678 |
| ANXA5 | 0.005460739 | 0.092301956 | 1.892260153 |
| XPO1 | 0.023287448 | 0.160212895 | 1.891644581 |
| RPL22L1 | 0.008634971 | 0.109651854 | 1.891148007 |
| CKS2 | 0.002563896 | 0.06668617 | 1.88738245 |
| NCOR1P1 | 0.003229218 | 0.073731518 | 1.88301663 |
| PITPNB | 0.028179466 | 0.171837495 | 1.878853456 |
| MAPKAPK5-AS1 | 0.009399038 | 0.11359998 | 1.874808729 |
| CLDND2 | 5.23E-06 | 0.002432951 | 1.874552952 |
| MS4A6A | 0.012095817 | 0.125703509 | 1.869808769 |
| SCPEP1 | 0.000686051 | 0.036431485 | 1.8668755 |
| CDK1 | 0.000684486 | 0.036431485 | 1.864968897 |
| GPR62 | 0.027714941 | 0.170799695 | 1.859738439 |
| KIF14 | 0.000211756 | 0.020194974 | 1.857972072 |
| KIAA1524 | 0.000129673 | 0.015807959 | 1.856687697 |
| DLEU2 | 0.032606313 | 0.181305647 | 1.852510613 |
| CD38 | 0.001742654 | 0.054233003 | 1.848127842 |
| MRPL13 | 0.000238247 | 0.021223109 | 1.847670465 |
| KIF21A | 0.000988107 | 0.042854541 | 1.847404049 |
| EPSTI1 | 0.014297007 | 0.13317809 | 1.842110764 |
| ATAD2 | 0.001459616 | 0.049801877 | 1.841932646 |
| MIR375 | 7.03E-05 | 0.011206925 | 1.8400243 |
| CCT6A | 0.016382995 | 0.141284174 | 1.83640635 |
| NUSAP1 | 0.002841531 | 0.069154677 | 1.835004444 |
| IGBP1P1 | 0.000924221 | 0.042067769 | 1.831605868 |
| RASA2 | 0.007074403 | 0.103501868 | 1.827742534 |
| FAM26F | 0.004044 | 0.081640892 | 1.826535913 |
| PRKAR1A | 0.027849707 | 0.171171149 | 1.826029809 |
| CHCHD3 | 0.002551805 | 0.066542354 | 1.822611374 |
| NDC80 | 0.001180286 | 0.046610434 | 1.822261972 |
| ARAP2 | 0.012911662 | 0.128376993 | 1.821819578 |
| DLGAP5 | 0.004600915 | 0.085469493 | 1.81963837 |
| MRPS23 | 0.000141799 | 0.01649964 | 1.819314705 |
| ENY2 | 0.017861311 | 0.146963978 | 1.819171633 |
| SERPINB1 | 0.030619644 | 0.176571726 | 1.818911413 |
| ARL8B | 0.00137571 | 0.049200144 | 1.816284576 |
| CMC1 | 0.000673333 | 0.036167106 | 1.809407033 |
| BIRC5 | 1.23E-05 | 0.003737134 | 1.809271706 |
| SKA3 | 0.002304848 | 0.063715009 | 1.806265775 |
| HMMR | 8.93E-06 | 0.003344015 | 1.805751926 |
| POSTN | 0.000971746 | 0.042439677 | 1.804176594 |
| FAM111B | 0.002232954 | 0.062608579 | 1.803835219 |
| NCF2 | 3.47E-05 | 0.006749148 | 1.803809037 |
| ATP5F1 | 0.043535702 | 0.201232112 | 1.802550517 |
| CEP128 | 0.005621419 | 0.093043648 | 1.801996528 |
| SLC30A1 | 0.000963256 | 0.042439677 | 1.800382925 |
| SMC2 | 0.004794421 | 0.086727852 | 1.798958088 |
| LOC105371371 | 0.008158575 | 0.108131647 | 1.798487131 |
| KNL1 | 0.000438963 | 0.029165497 | 1.797966775 |
| SAT1 | 0.041452342 | 0.197652993 | 1.794431135 |
| MGC39584 | 0.01419995 | 0.132762237 | 1.793648752 |
| RAB27A | 0.000591565 | 0.033745371 | 1.792505851 |
| CCL8 | 0.001561928 | 0.051557307 | 1.792502348 |
| CIDEB | 0.024598448 | 0.163591697 | 1.789399491 |
| C1QBP | 0.011856754 | 0.124325025 | 1.788928358 |
| AOAH | 0.019764467 | 0.151411257 | 1.788062299 |
| NMI | 0.00177176 | 0.054233003 | 1.787916465 |
| SUV39H2 | 0.000305984 | 0.024373052 | 1.784402292 |
| ZCCHC6 | 0.043091909 | 0.200221534 | 1.783489024 |
| STAT1 | 0.016922122 | 0.143561401 | 1.781503683 |
| RAP1B | 0.040571755 | 0.196739449 | 1.778926664 |
| HMGB2 | 0.013384797 | 0.129799151 | 1.776522429 |
| EXOSC7 | 0.004278259 | 0.082614182 | 1.776461258 |
| HSPH1 | 0.007131944 | 0.103636398 | 1.775629048 |
| PABPN1 | 0.030100161 | 0.175972089 | 1.770665437 |
| IFIH1 | 0.011041776 | 0.121223714 | 1.770258272 |
| ST20 | 0.001422925 | 0.049801877 | 1.770217667 |
| JAKMIP1 | 5.41E-06 | 0.002432951 | 1.76972642 |
| CCDC34 | 0.006575178 | 0.100632015 | 1.766762241 |
| TXNDC9 | 0.00656991 | 0.100632015 | 1.766250949 |
| TDG | 0.024360883 | 0.163563609 | 1.760241419 |
| MPHOSPH9 | 0.003485638 | 0.075013196 | 1.758496547 |
| GOLGA8EP | 0.031460708 | 0.178479704 | 1.75470176 |
| GOLGA6C | 0.03862728 | 0.194236448 | 1.753864449 |
| HLTF | 0.000358908 | 0.026063263 | 1.753768397 |
| MT2A | 0.04842162 | 0.20964602 | 1.753408854 |
| TICRR | 0.000171332 | 0.018196529 | 1.753115631 |
| LOC440867 | 0.007447499 | 0.104575811 | 1.752262995 |
| PCNA | 0.001570341 | 0.051557307 | 1.751670789 |
| NAMPT | 0.028700201 | 0.172810596 | 1.749531775 |
| FOXD2 | 0.009716073 | 0.11514105 | 1.748628039 |
| RAB8B | 0.033733619 | 0.182842631 | 1.748581823 |
| ANKHD1 | 0.040534052 | 0.196739449 | 1.747059492 |
| UTP3 | 0.00338542 | 0.074744712 | 1.745765319 |
| DPH3P1 | 0.003511107 | 0.075013196 | 1.744559422 |
| TYROBP | 0.013620131 | 0.130604473 | 1.742334169 |
| LLPH | 0.000292129 | 0.024279213 | 1.742035969 |
| KIF23 | 0.0001914 | 0.019789102 | 1.741353157 |
| MT1B | 0.000127521 | 0.015795908 | 1.740674062 |
| CDCA2 | 5.77E-05 | 0.009516077 | 1.739927675 |
| CD86 | 0.004825473 | 0.086822121 | 1.738844613 |
| SUZ12 | 0.04125358 | 0.197652993 | 1.738125705 |
| C18orf54 | 0.000120523 | 0.0157134 | 1.738008368 |
| EID1 | 0.000332555 | 0.025870261 | 1.737744309 |
| FASLG | 0.00149775 | 0.050221331 | 1.736526161 |
| SLAMF8 | 0.001002188 | 0.042897175 | 1.734632022 |
| IGSF6 | 0.005746074 | 0.094548749 | 1.734395484 |
| ANP32B | 0.0071956 | 0.103873303 | 1.733708078 |
| MT1G | 0.026656179 | 0.169018867 | 1.73343307 |
| FN1 | 0.016519518 | 0.142167535 | 1.73203254 |
| PSMA1 | 0.026748952 | 0.169071099 | 1.728976857 |
| LRRC8C | 0.012203595 | 0.12604406 | 1.726381724 |
| LCP2 | 0.016985677 | 0.143561401 | 1.726082606 |
| CELF2-AS1 | 0.022748963 | 0.158668126 | 1.724841588 |
| RPS10P19 | 0.047777346 | 0.2084661 | 1.723901887 |
| SPP1 | 0.01627209 | 0.14097153 | 1.722991471 |
| MND1 | 0.00409636 | 0.081689623 | 1.721574599 |
| KIAA0101 | 0.007448261 | 0.104575811 | 1.720769122 |
| IL13RA2 | 0.005911702 | 0.096188913 | 1.720457516 |
| FGF9 | 0.010064071 | 0.117100789 | 1.71899442 |
| RAP1A | 0.006138805 | 0.097354435 | 1.717216701 |
| SCRG1 | 0.016294365 | 0.14097153 | 1.715744056 |
| NELL2 | 0.011832155 | 0.12422387 | 1.713462525 |
| ZNHIT3 | 0.001957918 | 0.058401071 | 1.712927489 |
| ZNF845 | 0.031404151 | 0.178460482 | 1.712372449 |
| C19orf53 | 0.019088104 | 0.149381255 | 1.712189284 |
| NUP37 | 0.002249621 | 0.062824603 | 1.711565568 |
| OR6C74 | 0.001882074 | 0.056742423 | 1.71144209 |
| SFR1 | 0.000126075 | 0.015781052 | 1.710805554 |
| PILRA | 0.000168343 | 0.018015516 | 1.708016452 |
| HIST1H3J | 1.83E-05 | 0.004604994 | 1.708012281 |
| POLE2 | 0.000225203 | 0.020635117 | 1.705915317 |
| LAP3 | 0.015706547 | 0.138304184 | 1.705516856 |
| OR8H3 | 0.000358578 | 0.026063263 | 1.704751711 |
| TEX30 | 0.002815888 | 0.068965611 | 1.703903002 |
| MKI67 | 0.002418633 | 0.064832444 | 1.703714339 |
| CMC2 | 0.006880015 | 0.102391198 | 1.702568354 |
| UTP11 | 0.000432419 | 0.029060616 | 1.700976136 |
| FPR3 | 0.044992071 | 0.204061527 | 1.700849849 |
| PLA2G7 | 0.016347048 | 0.141119239 | 1.70044876 |
| CCL18 | 0.013352238 | 0.129799151 | 1.700121864 |
| PDP1 | 0.007295693 | 0.104575811 | 1.699255631 |
| HDAC2 | 0.015289985 | 0.136851203 | 1.69871021 |
| BORCS7 | 0.030099576 | 0.175972089 | 1.69858984 |
| CCNA2 | 0.000197711 | 0.019789102 | 1.698213702 |
| APOL4 | 1.75E-05 | 0.004604994 | 1.697414143 |
| GMNN | 0.000102727 | 0.014846871 | 1.697099501 |
| CD244 | 0.000459162 | 0.029949132 | 1.697003073 |
| MINPP1 | 0.004722628 | 0.086313559 | 1.694681419 |
| IFIT2 | 0.00412232 | 0.081725849 | 1.694240071 |
| HIST3H2BB | 0.000609596 | 0.034184262 | 1.694187876 |
| PCNX4 | 0.008329239 | 0.108936848 | 1.693713965 |
| PSMB9 | 0.00277476 | 0.068763784 | 1.693255397 |
| SNORD116-6 | 0.044832978 | 0.203798803 | 1.693034602 |
| ITM2A | 0.010440991 | 0.11785398 | 1.689846609 |
| PTGES3 | 0.047475548 | 0.208164953 | 1.68788005 |
| IWS1 | 0.009391866 | 0.11359998 | 1.686918053 |
| SEC11A | 0.000945832 | 0.042439677 | 1.686849995 |
| TNFSF13B | 0.013832396 | 0.131649375 | 1.685287941 |
| ISY1 | 0.036330647 | 0.188919764 | 1.684998857 |
| KLRG1 | 0.020097839 | 0.152355827 | 1.684329041 |
| ACSL1 | 0.000960736 | 0.042439677 | 1.684260813 |
| MED27 | 0.006927298 | 0.102626002 | 1.683640497 |
| PMCH | 0.003617245 | 0.076371963 | 1.68329619 |
| SPCS1 | 0.006051431 | 0.097010149 | 1.682419563 |
| AADACL3 | 0.000221961 | 0.020607445 | 1.681644199 |
| DMXL2 | 0.007908512 | 0.106504566 | 1.681279836 |
| KRTAP12-2 | 3.20E-05 | 0.006509799 | 1.680783851 |
| CENPN | 0.000155276 | 0.017276595 | 1.679102175 |
| SNORD77 | 0.004105404 | 0.081725849 | 1.678306652 |
| PSMA2 | 0.007764027 | 0.106039556 | 1.677745597 |
| MS4A6E | 0.00019836 | 0.019789102 | 1.67734145 |
| NASP | 0.010331723 | 0.117537008 | 1.675749083 |
| BRIP1 | 0.000158107 | 0.017292007 | 1.674872883 |
| OR1J1 | 0.005468386 | 0.092301956 | 1.674839602 |
| HIST1H2BD | 0.023358998 | 0.160212895 | 1.673884574 |
| HNRNPA2B1 | 0.030344738 | 0.176569528 | 1.672234489 |
| PPP2R2B | 0.000338783 | 0.025870261 | 1.671808104 |
| SNRPF | 0.006449349 | 0.099795707 | 1.670921843 |
| CCL7 | 0.00039569 | 0.0271925 | 1.670436867 |
| TFEC | 0.013296089 | 0.129769705 | 1.66914923 |
| APOBEC3C | 0.010362605 | 0.117537008 | 1.668950569 |
| CCT3 | 0.001650087 | 0.052906438 | 1.667940815 |
| C8orf59 | 0.045204604 | 0.204576005 | 1.667765703 |
| EBNA1BP2 | 0.000670473 | 0.036167106 | 1.666832859 |
| MIR103A2 | 0.003401551 | 0.074744712 | 1.666585345 |
| SUB1 | 0.024051105 | 0.162602529 | 1.66632617 |
| FAM159B | 0.00481274 | 0.086822121 | 1.666305225 |
| LOC339803 | 0.000542954 | 0.032253354 | 1.666252478 |
| DEPDC1B | 0.000840695 | 0.040641012 | 1.666119561 |
| HSD11B1 | 0.00023779 | 0.021223109 | 1.665251556 |
| CCDC26 | 0.00072574 | 0.0371044 | 1.665244331 |
| CHCHD1 | 0.000547238 | 0.032370709 | 1.665070957 |
| CSAG4 | 0.001762308 | 0.054233003 | 1.664917811 |
| INO80B | 0.032579225 | 0.181305647 | 1.664225253 |
| LYRM9 | 0.008794135 | 0.110769995 | 1.663147578 |
| C1orf56 | 0.039653873 | 0.196021419 | 1.661688218 |
| NDUFB3 | 0.001244725 | 0.047480916 | 1.661123015 |
| IFITM1 | 0.023988273 | 0.162451018 | 1.660419946 |
| SOAT1 | 0.001375714 | 0.049200144 | 1.659681644 |
| CTSC | 0.014475852 | 0.133786984 | 1.65819611 |
| CSE1L | 0.031301769 | 0.178151726 | 1.657117154 |
| GPR63 | 0.015932571 | 0.13916659 | 1.656930783 |
| FRG1 | 0.023938418 | 0.162438612 | 1.656448148 |
| APOBEC3B | 0.007824282 | 0.106186254 | 1.655132085 |
| PARP9 | 0.003996416 | 0.08131592 | 1.65494143 |
| SPRR2E | 0.00246184 | 0.065119054 | 1.654726792 |
| ATG3 | 0.020058597 | 0.152332653 | 1.653464066 |
| HSPA9 | 0.007082404 | 0.103501868 | 1.653380592 |
| APOPT1 | 0.011047579 | 0.121223714 | 1.65178065 |
| SET | 0.024840251 | 0.164661107 | 1.650885024 |
| ESM1 | 0.001304861 | 0.048101984 | 1.649248895 |
| RTCA | 0.009128789 | 0.113355701 | 1.648577434 |
| CD274 | 0.029291246 | 0.173758444 | 1.648143423 |
| ERMP1 | 0.000516636 | 0.031418657 | 1.645654008 |
| THAP5P1 | 0.001711895 | 0.053810409 | 1.643051093 |
| STAT4 | 0.027165805 | 0.170173771 | 1.642912735 |
| PSMD12 | 0.03790421 | 0.19288131 | 1.641689743 |
| CDC45 | 0.001147863 | 0.046365797 | 1.639659161 |
| FAM46D | 0.003121566 | 0.072333792 | 1.638622259 |
| ST8SIA4 | 0.016816554 | 0.143316193 | 1.638493767 |
| EAF1 | 0.004238939 | 0.082422549 | 1.637739853 |
| MCM6 | 0.00143173 | 0.049801877 | 1.636674353 |
| CDC6 | 0.000519703 | 0.031418657 | 1.635332001 |
| NAB1 | 0.002468426 | 0.065170291 | 1.633796342 |
| LNPK | 0.002531283 | 0.066474195 | 1.633195026 |
| MEDAG | 0.000792783 | 0.038860847 | 1.631684472 |
| MFAP1 | 0.002459731 | 0.065119054 | 1.630851869 |
| NCAPH | 0.000964245 | 0.042439677 | 1.628794558 |
| SUPT4H1 | 0.024306643 | 0.163355903 | 1.627758494 |
| DCAF4L1 | 0.016725101 | 0.142919495 | 1.627247296 |
| RABGGTB | 0.033370365 | 0.181841846 | 1.626503283 |
| KRTAP13-1 | 0.000135245 | 0.016345064 | 1.625779874 |
| C1QTNF9B | 0.011742726 | 0.124044656 | 1.62550732 |
| SLA | 0.007800208 | 0.106186254 | 1.625379964 |
| RNASEH2A | 1.61E-05 | 0.00442789 | 1.622192813 |
| DCAF4L2 | 0.002020675 | 0.059388496 | 1.621551018 |
| IL23R | 0.002167821 | 0.061598877 | 1.621062398 |
| ARFIP1 | 0.003006002 | 0.070707765 | 1.620344773 |
| MCTP2 | 0.002287085 | 0.063365952 | 1.619865853 |
| GADD45GIP1 | 0.011554576 | 0.123183461 | 1.618609752 |
| BLOC1S4 | 0.001126036 | 0.046158354 | 1.617593366 |
| KATNA1 | 0.007094919 | 0.103501868 | 1.617377116 |
| HDGFRP3 | 0.007840628 | 0.106202474 | 1.617262131 |
| CEP295 | 0.007550625 | 0.105030239 | 1.615807823 |
| OVOS2 | 0.040239283 | 0.196589278 | 1.615579892 |
| DRAM1 | 0.000592141 | 0.033745371 | 1.615452753 |
| DNA2 | 0.004118545 | 0.081725849 | 1.615285101 |
| MCM8 | 0.000852665 | 0.040916856 | 1.615104275 |
| ATP6V1D | 0.013302392 | 0.129769705 | 1.613491891 |
| NCL | 0.048478028 | 0.209678682 | 1.611752229 |
| ECT2 | 0.002846251 | 0.069154677 | 1.611379697 |
| ZNF733P | 0.028170979 | 0.171837495 | 1.610814996 |
| CCDC18 | 0.007449284 | 0.104575811 | 1.610706437 |
| PRR27 | 0.00235494 | 0.06385773 | 1.610225839 |
| C7orf25 | 0.010417679 | 0.117791026 | 1.610009693 |
| CENPH | 0.001313489 | 0.048204457 | 1.609715666 |
| LRRC70 | 0.000343619 | 0.025899291 | 1.609047195 |
| MIR9-1 | 0.013143674 | 0.129013216 | 1.608692946 |
| RFC4 | 0.003367486 | 0.074744712 | 1.608557729 |
| VN1R10P | 0.006038998 | 0.096978343 | 1.608234126 |
| CHORDC1 | 0.047544429 | 0.208164953 | 1.607676971 |
| TOPBP1 | 0.00987237 | 0.116109914 | 1.607531923 |
| PLSCR1 | 0.029300109 | 0.173758444 | 1.606911333 |
| HIST1H4F | 0.013331465 | 0.129789489 | 1.606228037 |
| HNRNPC | 0.039000756 | 0.194867383 | 1.606136987 |
| FANCI | 0.00158723 | 0.051919936 | 1.603429425 |
| CEP57L1 | 0.004598611 | 0.085469493 | 1.602191963 |
| HSPA13 | 0.002186942 | 0.061937863 | 1.601731946 |
| MRPS14 | 0.01284919 | 0.128050442 | 1.601725652 |
| GPR15 | 0.012632737 | 0.127640725 | 1.60154634 |
| SUMO1 | 0.026868074 | 0.169213699 | 1.601451578 |
| PGM2 | 0.002143353 | 0.061574086 | 1.601370367 |
| LSM6 | 0.023327673 | 0.160212895 | 1.600858804 |
| SRSF7 | 0.029754901 | 0.175211025 | 1.600477547 |
| UQCRBP1 | 0.027295341 | 0.17025725 | 1.599534166 |
| ARHGAP11B | 0.018837792 | 0.14886785 | 1.598975269 |
| GZMH | 0.000317799 | 0.025029784 | 1.597948106 |
| RACGAP1 | 0.000580036 | 0.033579811 | 1.597433223 |
| KIF20A | 0.000340631 | 0.025870261 | 1.597176233 |
| KRTAP15-1 | 0.032016269 | 0.179958606 | 1.596853447 |
| RPL39L | 0.007977668 | 0.106767116 | 1.595774606 |
| LMBR1 | 0.009239083 | 0.11359998 | 1.595348301 |
| LINS1 | 0.002542008 | 0.066486925 | 1.595292192 |
| TLR2 | 0.001215527 | 0.047020347 | 1.595060394 |
| MAK16 | 0.00077521 | 0.03826709 | 1.594613673 |
| PTENP1 | 0.016994457 | 0.143561401 | 1.59447344 |
| SLFN11 | 0.013090802 | 0.129013216 | 1.593850555 |
| NAT1 | 0.007646577 | 0.105510947 | 1.592397918 |
| SNRPG | 0.001035314 | 0.043586515 | 1.591646428 |
| TAF15 | 0.02054321 | 0.153374964 | 1.59149169 |
| RAB39B | 0.023250133 | 0.160092804 | 1.591430631 |
| DARS | 0.010227678 | 0.117355653 | 1.591058559 |
| KLHL8 | 0.002054268 | 0.059998438 | 1.590394966 |
| WDCP | 0.004155531 | 0.081854963 | 1.589718516 |
| N4BP2 | 0.041466129 | 0.197652993 | 1.589705726 |
| PSMA4 | 0.019042472 | 0.149306925 | 1.58921039 |
| COX19 | 0.011779487 | 0.124164913 | 1.588911949 |
| MCM3 | 0.006000441 | 0.096914245 | 1.587913452 |
| DNAJC21 | 0.021759032 | 0.155873717 | 1.587710007 |
| ALG6 | 0.041269776 | 0.197652993 | 1.585739344 |
| EXO1 | 0.000824031 | 0.039984165 | 1.585735269 |
| HIST3H2A | 0.001686336 | 0.053245797 | 1.585541902 |
| LOC100101478 | 0.020180628 | 0.152355827 | 1.585054252 |
| RCN2 | 0.001897691 | 0.057090484 | 1.584959435 |
| MSH2 | 0.00116044 | 0.046365797 | 1.584526221 |
| UPP1 | 0.001398147 | 0.04949734 | 1.584436128 |
| METAP1 | 0.040559854 | 0.196739449 | 1.584135337 |
| SNORD59B | 0.036357671 | 0.188990189 | 1.58357488 |
| AQP9 | 0.003528656 | 0.075066829 | 1.5829768 |
| RIPK2 | 0.040638944 | 0.1968526 | 1.582235581 |
| MCEE | 0.001130055 | 0.046188055 | 1.582128027 |
| COMMD8 | 0.005286041 | 0.091164 | 1.581720236 |
| CLEC4E | 0.000388081 | 0.02693362 | 1.581673222 |
| MRPL22 | 0.001761687 | 0.054233003 | 1.58161648 |
| UBE2V2 | 0.009050785 | 0.112893248 | 1.581301592 |
| MIR503HG | 0.009229247 | 0.11359998 | 1.581233112 |
| CDC7 | 0.000123113 | 0.0157134 | 1.580699591 |
| H2AFX | 0.002979937 | 0.070538535 | 1.580653324 |
| ATP13A3 | 0.028111123 | 0.171638735 | 1.579181288 |
| LIAS | 0.000683528 | 0.036431485 | 1.578802841 |
| CMSS1 | 0.010820485 | 0.119822168 | 1.577283989 |
| TRGV5 | 0.01093837 | 0.120660869 | 1.57725697 |
| SFT2D2 | 0.010254535 | 0.117355653 | 1.57701428 |
| TMEM68 | 0.000208377 | 0.020008832 | 1.575856295 |
| KIFC1 | 0.002448542 | 0.065012633 | 1.575688607 |
| FLVCR1-AS1 | 0.000597932 | 0.033800589 | 1.575513218 |
| AKAP5 | 0.014176339 | 0.132733196 | 1.575456078 |
| CEP55 | 0.007669102 | 0.105613859 | 1.575201318 |
| TRAPPC2 | 0.011356531 | 0.12228097 | 1.574939729 |
| GAFA2 | 0.021741321 | 0.155826465 | 1.573622948 |
| ACOT1 | 0.039011201 | 0.194867383 | 1.573488615 |
| SRI | 0.013148967 | 0.129013216 | 1.573483193 |
| HILPDA | 0.006068657 | 0.097010149 | 1.573421728 |
| TCTEX1D2 | 0.045806461 | 0.205558081 | 1.572993999 |
| RNF175 | 0.000489587 | 0.030591988 | 1.572639268 |
| IL12RB2 | 0.000824255 | 0.039984165 | 1.571210573 |
| TLK2 | 0.012191505 | 0.12604406 | 1.57111996 |
| KNTC1 | 0.024902418 | 0.164661107 | 1.570496413 |
| S100A11 | 0.032779867 | 0.181473716 | 1.5667293 |
| SMIM4 | 0.030580994 | 0.176569528 | 1.566337307 |
| SLC16A6 | 0.001217498 | 0.047020347 | 1.565542046 |
| RBBP8 | 0.034230914 | 0.184077884 | 1.565108748 |
| CTBS | 0.006474017 | 0.100066953 | 1.56415849 |
| RNF149 | 0.027142823 | 0.170173771 | 1.562455421 |
| CENPJ | 0.001191091 | 0.046773624 | 1.561669566 |
| FAM58A | 0.001354636 | 0.04869475 | 1.561547075 |
| NOB1 | 0.006270818 | 0.097861952 | 1.561240078 |
| PRKRA | 0.001335754 | 0.048383706 | 1.560502778 |
| DUSP12 | 0.005433915 | 0.092301956 | 1.560138959 |
| OMG | 0.001027236 | 0.043376688 | 1.559766949 |
| SMNDC1 | 0.043404035 | 0.201154092 | 1.559655979 |
| CCR5 | 0.000907136 | 0.041933291 | 1.55960926 |
| SYTL3 | 0.008453954 | 0.109623214 | 1.559527782 |
| LOC157740 | 0.032227039 | 0.180286616 | 1.559341693 |
| SERPING1 | 0.026762696 | 0.169081741 | 1.55884235 |
| CLDN17 | 0.003520784 | 0.075013196 | 1.558631371 |
| EED | 0.000660994 | 0.036167106 | 1.557988905 |
| RANBP2 | 0.044816868 | 0.203794344 | 1.55775268 |
| MRPS28 | 0.020753144 | 0.154028044 | 1.557639458 |
| FBXO22 | 0.002586195 | 0.067064188 | 1.557287639 |
| EID2B | 0.002040995 | 0.059735249 | 1.557252606 |
| UBLCP1 | 0.029788674 | 0.175291874 | 1.557159177 |
| TTF2 | 0.001624237 | 0.05263449 | 1.557074192 |
| BTN3A3 | 0.02624817 | 0.168334457 | 1.556717443 |
| DEFB134 | 0.000952418 | 0.042439677 | 1.55649127 |
| NUDT21 | 0.040754592 | 0.1968526 | 1.55645339 |
| FKBP5 | 0.026709368 | 0.169018867 | 1.555786218 |
| DHX9 | 0.006174674 | 0.097592073 | 1.555246698 |
| BCAP29 | 0.033847924 | 0.183142347 | 1.554697029 |
| METAP2 | 0.021263802 | 0.154629827 | 1.554059264 |
| C1orf54 | 0.017948906 | 0.146963978 | 1.553960558 |
| MIR101-1 | 0.000303633 | 0.024373052 | 1.553916753 |
| ASB14 | 0.048848285 | 0.210130509 | 1.553895386 |
| KRT86 | 0.022988085 | 0.159547015 | 1.553198202 |
| SPATA13 | 0.014631737 | 0.134332469 | 1.55205143 |
| CHEK1 | 0.038177597 | 0.193446716 | 1.551838309 |
| NAA10 | 0.001118444 | 0.04611685 | 1.550597023 |
| ZNF788 | 0.001321844 | 0.048258418 | 1.550439224 |
| HSPA4 | 0.011180911 | 0.122023591 | 1.550349732 |
| CXorf58 | 0.000767024 | 0.037996769 | 1.550228703 |
| LOC100288884 | 0.025611536 | 0.166478062 | 1.547891402 |
| BIVM | 0.0037466 | 0.077698875 | 1.54774935 |
| BUB1B | 0.001519827 | 0.050730487 | 1.547718723 |
| UTP18 | 0.006979421 | 0.103000358 | 1.547190633 |
| DEFB127 | 0.000296831 | 0.024349445 | 1.547113151 |
| LOC100131860 | 0.018348222 | 0.147681707 | 1.546413261 |
| NEIL3 | 0.001208755 | 0.046941314 | 1.546229563 |
| ATAD5 | 0.00997208 | 0.116890366 | 1.544571366 |
| RNF213 | 0.029965629 | 0.17577198 | 1.543823337 |
| CST7 | 0.000519938 | 0.031418657 | 1.543508775 |
| TRGV7 | 0.001331645 | 0.048378372 | 1.542821929 |
| HIST1H3F | 0.026819557 | 0.169202121 | 1.542498437 |
| OR4D11 | 0.019387366 | 0.150082174 | 1.541599721 |
| PDCD1LG2 | 0.000722863 | 0.0371044 | 1.540720539 |
| PNMA1 | 0.001462602 | 0.049801877 | 1.540697942 |
| LARS2 | 0.005318459 | 0.091485516 | 1.540457791 |
| PDHB | 0.011490406 | 0.122983367 | 1.539790186 |
| DCLRE1B | 0.033207955 | 0.181571744 | 1.539190488 |
| PSMD1 | 0.025987313 | 0.167552058 | 1.538947822 |
| HACD3 | 0.002437292 | 0.064905131 | 1.538711004 |
| TOX | 0.015122068 | 0.136572761 | 1.538589765 |
| UCHL3 | 0.002699524 | 0.067826348 | 1.538131951 |
| ITGB1BP1 | 0.002996125 | 0.070593888 | 1.537391907 |
| RUVBL1 | 0.004615119 | 0.085469493 | 1.536589972 |
| RNU5F-1 | 0.018645275 | 0.148482306 | 1.536478842 |
| H2AFZ | 0.031471189 | 0.178479704 | 1.536061063 |
| SLF1 | 0.002219791 | 0.062608579 | 1.536046025 |
| RDX | 0.009358604 | 0.11359998 | 1.535940198 |
| FAM122B | 0.01134016 | 0.12228097 | 1.535899267 |
| MFSD14B | 0.015652183 | 0.138007265 | 1.53531436 |
| NKAP | 0.011801848 | 0.12422387 | 1.53461993 |
| CENPU | 0.005394546 | 0.09222852 | 1.534341457 |
| HPDL | 0.001711144 | 0.053810409 | 1.533827779 |
| PHLDA1 | 0.021810128 | 0.156159957 | 1.531348814 |
| HIST4H4 | 0.018420886 | 0.147822911 | 1.530271277 |
| CDK2 | 0.004051898 | 0.081640892 | 1.530257724 |
| C1QTNF9 | 0.039782696 | 0.19621037 | 1.530208646 |
| LOC151121 | 0.009433133 | 0.113697334 | 1.529867565 |
| RNASE10 | 0.001019119 | 0.043294751 | 1.529549034 |
| HYLS1 | 0.012095223 | 0.125703509 | 1.529178269 |
| PRKXP1 | 0.027323634 | 0.170265997 | 1.528789768 |
| IL1A | 0.000729059 | 0.0371044 | 1.528656717 |
| PRKDC | 0.026805571 | 0.169200117 | 1.527524833 |
| XPOT | 0.038778679 | 0.194419366 | 1.527024642 |
| XRCC2 | 0.011808739 | 0.12422387 | 1.526753858 |
| ACOT8 | 0.017957089 | 0.146963978 | 1.52616826 |
| PRIM2 | 0.002192223 | 0.061962255 | 1.525827595 |
| ICE1 | 0.005092149 | 0.089235016 | 1.525420096 |
| KIAA2026 | 0.045377158 | 0.205012182 | 1.525010091 |
| MNDA | 0.016654587 | 0.142629495 | 1.524948732 |
| WSB1 | 0.033890475 | 0.18323114 | 1.52365731 |
| SLC39A6 | 0.000367658 | 0.026294033 | 1.523359259 |
| GTSF1 | 0.042665873 | 0.199478309 | 1.523151609 |
| DEFB131 | 0.039151216 | 0.194968925 | 1.522897349 |
| SGO2 | 0.002079871 | 0.060244186 | 1.521925413 |
| SLC35G3 | 0.009308507 | 0.11359998 | 1.521144426 |
| MCM4 | 0.001138866 | 0.046278325 | 1.521073229 |
| XPO5 | 0.012253759 | 0.12604406 | 1.520347411 |
| HTR2B | 0.013415196 | 0.12983596 | 1.519319181 |
| EXOSC9 | 0.028780698 | 0.172810596 | 1.518195718 |
| OR2B6 | 0.004897957 | 0.087472088 | 1.517184836 |
| HIST2H2AC | 0.003377328 | 0.074744712 | 1.516909125 |
| FMNL2 | 0.018084673 | 0.147260535 | 1.516495331 |
| DPY19L2 | 0.04031512 | 0.196685648 | 1.516473773 |
| KIF18A | 0.006055572 | 0.097010149 | 1.516175884 |
| PTGDR | 0.005055172 | 0.088809048 | 1.515582578 |
| TREML3P | 0.001295718 | 0.048101984 | 1.515362496 |
| PPRC1 | 0.003487028 | 0.075013196 | 1.515137292 |
| SEPT7P9 | 0.011940376 | 0.124735372 | 1.515092662 |
| GNAI3 | 0.01034097 | 0.117537008 | 1.514849772 |
| SH2D2A | 0.016815969 | 0.143316193 | 1.514847488 |
| FAM102B | 0.010540744 | 0.118256765 | 1.514420147 |
| FANCB | 0.000199116 | 0.019789102 | 1.513069704 |
| BCAT1 | 0.023223887 | 0.160092804 | 1.512879727 |
| CHPF2 | 0.002811579 | 0.068965611 | 1.511748975 |
| HMGXB4 | 0.005528107 | 0.092334068 | 1.511515296 |
| GPN1 | 0.013418675 | 0.12983596 | 1.510976214 |
| MIR33A | 0.012360793 | 0.126303789 | 1.510767562 |
| SLC4A10 | 0.001122147 | 0.046133832 | 1.51046257 |
| PCDHGA7 | 0.013564169 | 0.130306914 | 1.510096294 |
| PARPBP | 0.008337779 | 0.108936848 | 1.509982588 |
| MRPS15 | 0.035043889 | 0.18561565 | 1.509501835 |
| MELK | 0.004133153 | 0.081725849 | 1.50926655 |
| OR4F13P | 0.000936208 | 0.042338433 | 1.508479449 |
| C3orf80 | 0.002778104 | 0.068763784 | 1.508304211 |
| ATP6V1A | 0.003686785 | 0.077431468 | 1.508274554 |
| ARMCX4 | 0.02624254 | 0.168334457 | 1.508164309 |
| CLIP4 | 0.015548747 | 0.137527728 | 1.507589288 |
| ORC6 | 0.002702818 | 0.067826348 | 1.507148185 |
| ERI1 | 0.013039251 | 0.128958394 | 1.507032384 |
| TBC1D31 | 0.029038516 | 0.173140275 | 1.507009495 |
| TMEM167A | 0.015989641 | 0.139471129 | 1.506648008 |
| SLC16A1 | 0.002607334 | 0.067223869 | 1.506270627 |
| RBPJ | 0.007818165 | 0.106186254 | 1.50576568 |
| PPA1 | 0.039061126 | 0.194867383 | 1.505617256 |
| ANLN | 0.006571501 | 0.100632015 | 1.505064008 |
| HOXB7 | 0.003518522 | 0.075013196 | 1.50454401 |
| FARSB | 0.008054963 | 0.107649554 | 1.504438043 |
| SFMBT2 | 0.022129843 | 0.157004056 | 1.504287439 |
| INHBA | 0.013905154 | 0.131898528 | 1.502043443 |
| TREX1 | 0.005460738 | 0.092301956 | 1.500326475 |
| DDX21 | 0.013778531 | 0.131315016 | 1.500124413 |
| ALG8 | 0.049493875 | 0.211286845 | 1.500041205 |

Supplmentary Methods

Gene expression profiling done with Affymetrix HuGene v1.0 st chips were first processed with frozen robust multiarray analysis (fRMA) algorithm to account for heterogeneity due to regional and temporal diversity in sampling and handling specimens.[[9](#_ENREF_9)] The probeset-by-probeset data were then transformed into gene-by-gene data by taking median intensity of probesets corresponding to a gene. For this, we chose probesets whose median intensities were larger than the half of maximum median intensity of probesets for the gene. Differentially expressed genes between extranodal and lymph node specimens were produced using the significance analysis of microarrays (SAM) algorithm.[[10](#_ENREF_10)]

**References**

1. Siu, L.L.; Wong, K.F.; Chan, J.K.; Kwong, Y.L. Comparative genomic hybridization analysis of natural killer cell lymphoma/leukemia. Recognition of consistent patterns of genetic alterations. *The American journal of pathology* **1999**, *155*, 1419-1425.

2. Siu, L.L.; Chan, V.; Chan, J.K.; Wong, K.F.; Liang, R.; Kwong, Y.L. Consistent patterns of allelic loss in natural killer cell lymphoma. *The American journal of pathology* **2000**, *157*, 1803-1809.

3. Ko, Y.H.; Choi, K.E.; Han, J.H.; Kim, J.M.; Ree, H.J. Comparative genomic hybridization study of nasal-type nk/t-cell lymphoma. *Cytometry* **2001**, *46*, 85-91.

4. Nakashima, Y.; Tagawa, H.; Suzuki, R.; Karnan, S.; Karube, K.; Ohshima, K.; Muta, K.; Nawata, H.; Morishima, Y.; Nakamura, S.*, et al.* Genome-wide array-based comparative genomic hybridization of natural killer cell lymphoma/leukemia: Different genomic alteration patterns of aggressive nk-cell leukemia and extranodal nk/t-cell lymphoma, nasal type. *Genes, chromosomes & cancer* **2005**, *44*, 247-255.

5. Iqbal, J.; Kucuk, C.; Deleeuw, R.J.; Srivastava, G.; Tam, W.; Geng, H.; Klinkebiel, D.; Christman, J.K.; Patel, K.; Cao, K.*, et al.* Genomic analyses reveal global functional alterations that promote tumor growth and novel tumor suppressor genes in natural killer-cell malignancies. *Leukemia* **2009**, *23*, 1139-1151.

6. Huang, Y.; de Reynies, A.; de Leval, L.; Ghazi, B.; Martin-Garcia, N.; Travert, M.; Bosq, J.; Briere, J.; Petit, B.; Thomas, E.*, et al.* Gene expression profiling identifies emerging oncogenic pathways operating in extranodal nk/t-cell lymphoma, nasal type. *Blood* **2010**, *115*, 1226-1237.

7. Sun, L.; Li, M.; Huang, X.; Xu, J.; Gao, Z.; Liu, C. High-resolution genome-wide analysis identified recurrent genetic alterations in nk/t-cell lymphoma, nasal type, which are associated with disease progression. *Medical oncology* **2014**, *31*, 71.

8. Ng, S.B.; Chung, T.H.; Kato, S.; Nakamura, S.; Takahashi, E.; Ko, Y.H.; Khoury, J.D.; Yin, C.C.; Soong, R.; Jeyasekharan, A.D.*, et al.* Epstein-barr virus-associated primary nodal t/nk-cell lymphoma shows a distinct molecular signature and copy number changes. *Haematologica* **2018**, *103*, 278-287.

9. McCall, M.N.; Bolstad, B.M.; Irizarry, R.A. Frozen robust multiarray analysis (frma). *Biostatistics* **2010**, *11*, 242-253.

10. Tusher, V.G.; Tibshirani, R.; Chu, G. Significance analysis of microarrays applied to the ionizing radiation response. *Proceedings of the National Academy of Sciences of the United States of America* **2001**, *98*, 5116-5121.
